# Pharmacological treatment options for cognitive dysfunction induced by multiple sclerosis: a network meta-analysis

**DOI:** 10.3389/fneur.2025.1649429

**Published:** 2025-10-07

**Authors:** Zhuyuan Fan, Dan Liu, Guangwei Zhang, Xinlin Guo

**Affiliations:** Department of Neurology, The First Affiliated Hospital of Baotou Medical College, Baotou, Mongolia, China

**Keywords:** multiple sclerosis, cognitive dysfunction, pharmacological intervention, randomized controlled trial, meta-analysis

## Abstract

**Objectives:**

To compare the effects of various pharmacological treatments on partial test results and adverse effects in patients with cognitive dysfunction (CD) induced by multiple sclerosis (MS) through a network meta-analysis.

**Methods:**

PubMed, Embase, Cochrane Library, and Web of Science databases were systematically retrieved for randomized controlled trials (RCTs) evaluating the influence of different pharmacological treatments on CD in MS patients. The search was updated until October 8, 2024. The Risk of Bias tool was used to assess the quality of eligible studies, and R was employed for data analysis.

**Results:**

Twenrt six studies involving 23,839 MS patients were included for our analysis. Network meta-analysis results indicated that compared to placebo, L-amphetamine may improve memory in MS-induced CD. Memantine may enhance performance on the Paced Auditory Serial Addition Test (PASAT). Compared to memantine, fampridine-SR, ginkgo biloba, and melatonin showed inferior effects. Atomoxetine may improve Symbol Digit Modalities Test (SDMT) scores, outperforming donepezil, ginkgo biloba, L-amphetamine, modafinil, and rivastigmine. Additionally, atomoxetine may improve California Verbal Learning Test (CVLT) performance, compared to ginkgo biloba, L-amphetamine, and memantine. In terms of adverse effects, rivastigmine was less likely to cause dyspepsia.

**Conclusion:**

Based on current evidence, L-amphetamine may improve memory in MS-induced CD. Melatonin may enhance PASAT performance, and atomoxetine may improve both SDMT and CVLT scores in these patients. However, rivastigmine was found to have a lower likelihood of causing dyspepsia among the treatments assessed.

**Systematic review registration:**

https://www.crd.york.ac.uk/PROSPERO/, identifier CRD42024623642.

## 1 Introduction

Multiple sclerosis (MS) is a chronic autoimmune, demyelinating, and neurodegenerative disease of the central nervous system (CNS) (1). Its clinical onset typically occurs between the ages of 20 and 40, making it the second most common cause of permanent disability in young adults (2). The incidence and prevalence of MS vary by region, with approximately 1 million people affected in America and 2.8 million individuals globally (3).

Throughout the course of MS, various neurological symptoms manifest depending on the specific neurons affected, with cognitive dysfunction (CD) being a common feature (4).

The prevalence of cognitive impairment in MS varies across the lifespan, with trajectories that may involve insidious, progressive decline or abrupt deterioration during disease relapses. Notably, this pattern of rapid decline has only been documented in recent years. Cognitive impairment, as a salient clinical feature of MS, has gained increasing recognition and is observed across all MS subtypes, including clinically isolated syndrome, early relapsing–remitting phases, and even radiologically isolated syndrome. These findings suggest that cognitive impairment may precede the clinical manifestations of the disease (5).

Cognitive impairments, which include both self-reported complaints and objective deficits, often present as functional alterations in brain networks, including changes in neuronal development, functional connectivity, and network integration (6). Among the most prevalent cognitive deficits are slowed processing speed and impaired working memory (7), which significantly impact daily life.

Previous studies have suggested that disease-modifying therapies (DMTs) for MS may be conducive to cognitive function, as these treatments primarily aim to halt disease progression and avoid relapses. However, whether they directly improve cognitive abilities is not clarified. There is evidence proving that DMTs may have positive effects on cognition, such as reducing T2 and T1 brain lesions, slowing brain atrophy, and inhibiting inflammatory activity in MS. Nevertheless, little data demonstrate the effectiveness of DMTs in treating CD associated with MS, and no medication has been authorized expressly for this use. Crucially, cognition has not been given priority as a primary outcome measure in first- or second-line DMT trials. Only several randomized controlled trials (RCTs) of DMTs have delved into cognitive outcomes more thoroughly, with conflicting results from symptomatic treatments like modafinil, donepezil, L-amphetamine sulfate, and memantine. Dalfampridine is a potential therapeutic option for MS-associated CD owing to its positive impact on gait; however, evidence regarding its effects on cognition remains inconsistent. One RCT reported significant, albeit transient, increases in processing speed, while another found no impact on processing speed. Despite the modest effects, statistically significant results were reported. To approve any drug for MS-related CD treatment, appropriate RCTs must be conducted with cognition as a primary endpoint (8).

A study by Gromisch et al. (9) showed that over 39% of participants reported that the most commonly prescribed medications at their clinics included anti-fatigue agents (86.4%), followed by stimulants (77.3%), disease-modifying therapies (45.5%), dopamine agonist antidepressants (40.9%), cholinesterase inhibitors (40.9%), and glutamate modulators (31.8%). In comparison to those in 2010, there was a decrease in the proportion of participants favoring cholinesterase inhibitors, a reduction in prescriptions for glutamate modulators, and an increase in stimulant prescriptions.

Clinical neuropsychologists employ more targeted and sensitive assessments, among which the Symbol Digit Modalities Test (SDMT) has become the gold standard for evaluating cognitive function in patients with MS.

Episodic memory tests focus on assessing learning across successive trials, followed by evaluation of memory retention or delayed recall after 20–30 min. The verbal memory assessment most commonly applied, the California Verbal Learning Test (CVLT), has been demonstrated to effectively discriminate cognitive impairment in patients with MS from that in healthy controls (10).

The Paced Auditory Serial Addition Test (PASAT) represents a key component of the Minimal Assessment of Cognitive Function in MS. This test is simple, efficient, and can be readily administered at the bedside within a short period, with the primary objective of evaluating sustained auditory attention and information processing speed. It constitutes a critical measure of cognitive function in MS (11)

Based on the studies included in our analysis, the final assessment tools selected were: memory tests (Memory), PASAT, SDMT, and CVLT. Therefore, these measures serve as the primary study outcomes in this work.

The pharmacological treatment options for MS-induced CD are diverse, and there is a lack of direct comparative studies. A network meta-analysis approach has been employed to conduct indirect comparisons among these treatments, aiming to identify which cognitive-enhancing medications effectively treat CD in the progressive MS cohort, as well as to assess the potential synergistic effects of combined drug therapies. This analysis offers new therapeutic options for treating this patient population.

## 2 Methods and materials

### 2.1 Literature search

A computerized search was carried out across the Cochrane, PubMed, Embase, as well as Web of Science databases for RCTs evaluating the effects of various medications on memory, adverse reactions, Paced Auditory Serial Addition Test (PASAT), Symbol Digit Modalities Test (SDMT), California Verbal Learning Test (CVLT), and related cognitive outcomes in MS patients. The search was conducted up to October 8, 2024. A combination of subject and free-text terms, including “MS,” “CD,” “Sclerosis, Multiple,” “MS,” “Sclerosis, Disseminated,” “Disseminated Sclerosis,” “MS, Acute Fulminating,” “CDs,” “Dysfunction, Cognitive,” “Dysfunctions, Cognitive,” and “Cognitive Disorder” “cognitive impairment” was used. Detailed search strategies are provided in Table 1 (with PubMed as an example) (Supplementary material 1).

**Table 1:** With PubMed as an example.docx.

Search strategy Pubmed (((multiple sclerosis[MeSH Terms]) OR (((((multiple sclerosis[Title/Abstract]) OR (MS[Title/Abstract])) OR (Sclerosis, Disseminated[Title/Abstract])) OR (Disseminated Sclerosis[Title/Abstract])) OR (Multiple Sclerosis, Acute Fulminating[Title/Abstract]))) AND ((cognitive dysfunction[MeSH Terms]) OR (((((((((((((((((((((((((Cognitive Dysfunctions[Title/Abstract]) OR (Dysfunction, Cognitive[Title/Abstract])) OR (Dysfunctions, Cognitive[Title/Abstract])) OR (Cognitive Disorder[Title/Abstract])) OR (Cognitive Disorders[Title/Abstract])) OR (Disorder, Cognitive[Title/Abstract])) OR (Disorders, Cognitive[Title/Abstract])) OR (Cognitive Impairments[Title/Abstract])) OR (Cognitive Impairment[Title/Abstract])) OR (Impairment, Cognitive[Title/Abstract])) OR (Impairments, Cognitive[Title/Abstract])) OR (Mild Cognitive Impairment[Title/Abstract])) OR (Cognitive Impairment, Mild[Title/Abstract])) OR (Cognitive Impairments, Mild[Title/Abstract])) OR (Impairment, Mild Cognitive[Title/Abstract])) OR (Impairments, Mild Cognitive[Title/Abstract])) OR (Mild Cognitive Impairments[Title/Abstract])) OR (Cognitive Decline[Title/Abstract])) OR (Cognitive Declines[Title/Abstract])) OR (Decline, Cognitive[Title/Abstract])) OR (Declines, Cognitive[Title/Abstract])) OR (Mental Deterioration[Title/Abstract])) OR (Deterioration, Mental[Title/Abstract])) OR (Deteriorations, Mental[Title/Abstract])) OR (Mental Deteriorations[Title/Abstract])))) AND (randomized controlled trial[Publication Type] OR randomized[Title/Abstract] OR placebo[Title/Abstract])
randomized controlled trial[Publication Type] OR randomized[Title/Abstract] OR placebo[Title/Abstract]
((multiple sclerosis[MeSH Terms]) OR (((((multiple sclerosis[Title/Abstract]) OR (MS[Title/Abstract])) OR (Sclerosis, Disseminated[Title/Abstract])) OR (Disseminated Sclerosis[Title/Abstract])) OR (Multiple Sclerosis, Acute Fulminating[Title/Abstract]))) AND ((cognitive dysfunction[MeSH Terms]) OR (((((((((((((((((((((((((Cognitive Dysfunctions[Title/Abstract]) OR (Dysfunction, Cognitive[Title/Abstract])) OR (Dysfunctions, Cognitive[Title/Abstract])) OR (Cognitive Disorder[Title/Abstract])) OR (Cognitive Disorders[Title/Abstract])) OR (Disorder, Cognitive[Title/Abstract])) OR (Disorders, Cognitive[Title/Abstract])) OR (Cognitive Impairments[Title/Abstract])) OR (Cognitive Impairment[Title/Abstract])) OR (Impairment, Cognitive[Title/Abstract])) OR (Impairments, Cognitive[Title/Abstract])) OR (Mild Cognitive Impairment[Title/Abstract])) OR (Cognitive Impairment, Mild[Title/Abstract])) OR (Cognitive Impairments, Mild[Title/Abstract])) OR (Impairment, Mild Cognitive[Title/Abstract])) OR (Impairments, Mild Cognitive[Title/Abstract])) OR (Mild Cognitive Impairments[Title/Abstract])) OR (Cognitive Decline[Title/Abstract])) OR (Cognitive Declines[Title/Abstract])) OR (Decline, Cognitive[Title/Abstract])) OR (Declines, Cognitive[Title/Abstract])) OR (Mental Deterioration[Title/Abstract])) OR (Deterioration, Mental[Title/Abstract])) OR (Deteriorations, Mental[Title/Abstract])) OR (Mental Deteriorations[Title/Abstract])))
(cognitive dysfunction[MeSH Terms]) OR (((((((((((((((((((((((((Cognitive Dysfunctions[Title/Abstract]) OR (Dysfunction, Cognitive[Title/Abstract])) OR (Dysfunctions, Cognitive[Title/Abstract])) OR (Cognitive Disorder[Title/Abstract])) OR (Cognitive Disorders[Title/Abstract])) OR (Disorder, Cognitive[Title/Abstract])) OR (Disorders, Cognitive[Title/Abstract])) OR (Cognitive Impairments[Title/Abstract])) OR (Cognitive Impairment[Title/Abstract])) OR (Impairment, Cognitive[Title/Abstract])) OR (Impairments, Cognitive[Title/Abstract])) OR (Mild Cognitive Impairment[Title/Abstract])) OR (Cognitive Impairment, Mild[Title/Abstract])) OR (Cognitive Impairments, Mild[Title/Abstract])) OR (Impairment, Mild Cognitive[Title/Abstract])) OR (Impairments, Mild Cognitive[Title/Abstract])) OR (Mild Cognitive Impairments[Title/Abstract])) OR (Cognitive Decline[Title/Abstract])) OR (Cognitive Declines[Title/Abstract])) OR (Decline, Cognitive[Title/Abstract])) OR (Declines, Cognitive[Title/Abstract])) OR (Mental Deterioration[Title/Abstract])) OR (Deterioration, Mental[Title/Abstract])) OR (Deteriorations, Mental[Title/Abstract])) OR (Mental Deteriorations[Title/Abstract]))
((((((((((((((((((((((((Cognitive Dysfunctions[Title/Abstract]) OR (Dysfunction, Cognitive[Title/Abstract])) OR (Dysfunctions, Cognitive[Title/Abstract])) OR (Cognitive Disorder[Title/Abstract])) OR (Cognitive Disorders[Title/Abstract])) OR (Disorder, Cognitive[Title/Abstract])) OR (Disorders, Cognitive[Title/Abstract])) OR (Cognitive Impairments[Title/Abstract])) OR (Cognitive Impairment[Title/Abstract])) OR (Impairment, Cognitive[Title/Abstract])) OR (Impairments, Cognitive[Title/Abstract])) OR (Mild Cognitive Impairment[Title/Abstract])) OR (Cognitive Impairment, Mild[Title/Abstract])) OR (Cognitive Impairments, Mild[Title/Abstract])) OR (Impairment, Mild Cognitive[Title/Abstract])) OR (Impairments, Mild Cognitive[Title/Abstract])) OR (Mild Cognitive Impairments[Title/Abstract])) OR (Cognitive Decline[Title/Abstract])) OR (Cognitive Declines[Title/Abstract])) OR (Decline, Cognitive[Title/Abstract])) OR (Declines, Cognitive[Title/Abstract])) OR (Mental Deterioration[Title/Abstract])) OR (Deterioration, Mental[Title/Abstract])) OR (Deteriorations, Mental[Title/Abstract])) OR (Mental Deteriorations[Title/Abstract])
cognitive dysfunction[MeSH Terms]
(multiple sclerosis[MeSH Terms]) OR (((((multiple sclerosis[Title/Abstract]) OR (MS[Title/Abstract])) OR (Sclerosis, Disseminated[Title/Abstract])) OR (Disseminated Sclerosis[Title/Abstract])) OR (Multiple Sclerosis, Acute Fulminating[Title/Abstract]))
((((multiple sclerosis[Title/Abstract]) OR (MS[Title/Abstract])) OR (Sclerosis, Disseminated[Title/Abstract])) OR (Disseminated Sclerosis[Title/Abstract])) OR (Multiple Sclerosis, Acute Fulminating[Title/Abstract])
multiple sclerosis[MeSH Terms]

### 2.2 Eligibility criteria

Studies were selected if they satisfied the following criteria: (1) Participants: Adults diagnosed with MS; (2) Interventions: Rivastigmine, 4-aminopyridine (4-AP), donepezil, ginkgo biloba, memantine, modafinil, methylphenidate, L-amphetamine, simvastatin, fampridine-SR, dalfampridine, CorSeNs, atomoxetine, lutein; (3) Control Group: Placebo; (4) Study Type: RCTs; (5) Outcome Measures: Memory, PASAT, SDMT, CVLT, and adverse reactions. Studies were excluded based on the criteria as follows: (1) Duplicate publications; (2) Animal studies, case reports, conference abstracts, reviews, protocols, or studies with incomplete or unavailable full-text data; (3) Studies involving other organic diseases or comorbidities.

### 2.3 Data extraction

Two authors independently screened the literature using EndNote based on the predefined eligibility criteria. Duplicate papers were eliminated, and a preliminary screening was performed. Titles and abstracts of the rest were reviewed to remove those failing to meet the eligibility criteria, followed by a re-screening process. At last, the full texts of the remaining studies were read for our final selection, and the results were compared. Dissents were addressed through discussion or by consulting a third party for consensus. Key data extracted from the eligible studies were: (1) first author, (2) year of publication, (3) country, (4) sample size, (5) gender, (6) age, (7) intervention, and (8) outcome indicators.

### 2.4 Quality assessment

The risk of bias was assessed as per the latest recommendations from the Cochrane Risk of Bias Tool 2.0 (12), covering the following seven domains: (1) random sequence generation; (2) allocation concealment; (3) blinding of participants and personnel; (4) blinding of outcome assessment; (5) incomplete outcome data; (6) selective reporting; (7) other sources of bias.

### 2.5 Data analysis

Bayesian network meta-analysis was undertaken with the help of R 4.4.1 (R Foundation for Statistical Computing) with a random-effects model for multiple treatment comparisons. Markov Chain Monte Carlo (MCMC) methods (13) were utilized to obtain the best combined estimates and probabilities for every treatment regimen. Convergence of the model was evaluated using trace plots and Brooks–Gelman–Rubin plots. Continuous outcomes were reported as mean differences (MD) with 95% confidence intervals (CI). The cumulative ranking of interventions was estimated using the Surface Under the Cumulative Ranking Curve (SUCRA) to examine the probability of every treatment being the most effective. Network and funnel plots were generated using STATA 15.0, with a direct macro command loaded. In the network plot, each node corresponds to a treatment being compared. The node size is proportional to the participant number in that treatment arm. Cumulative probability plots were produced through the ggplot2 package. SUCRA rankings were calculated in Stata software and used as the standard to evaluate the effectiveness of pharmacological interventions, with values of 0–1, where a higher value indicates better effectiveness. A funnel plot was generated to detect possible publication bias.

## 3 Results

### 3.1 Data screening process and results

**Figure 1:**
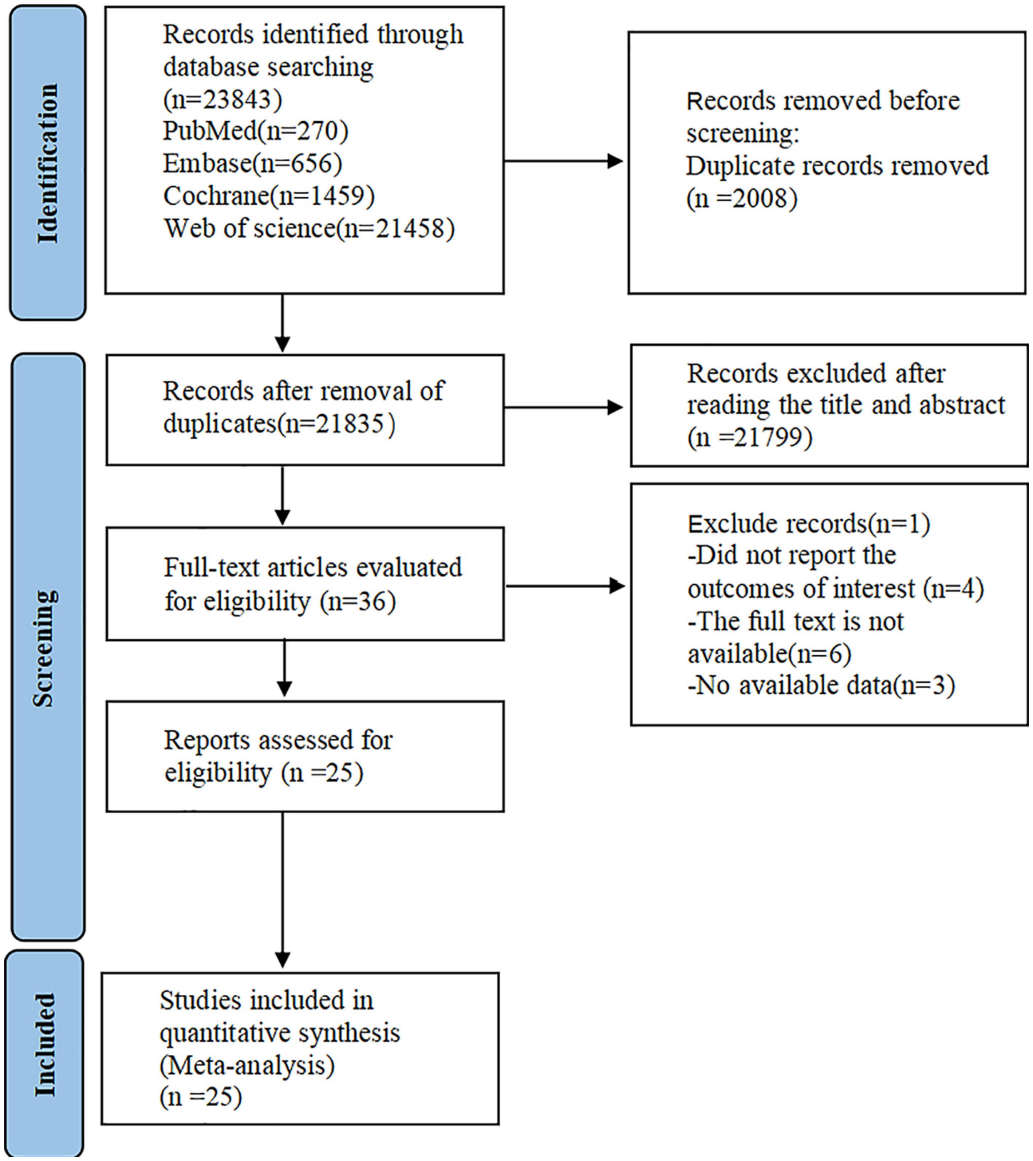
Flow chart of literature selection.

A preliminary search yielded 23,839 articles. After removing 1,004 duplicate articles, 21,799 articles were ostracized after title and abstract review. Eleven articles were removed after full-text evaluation. Ultimately, 25 articles (14–38) were eligible (Figure 1).

**Figure 2:**
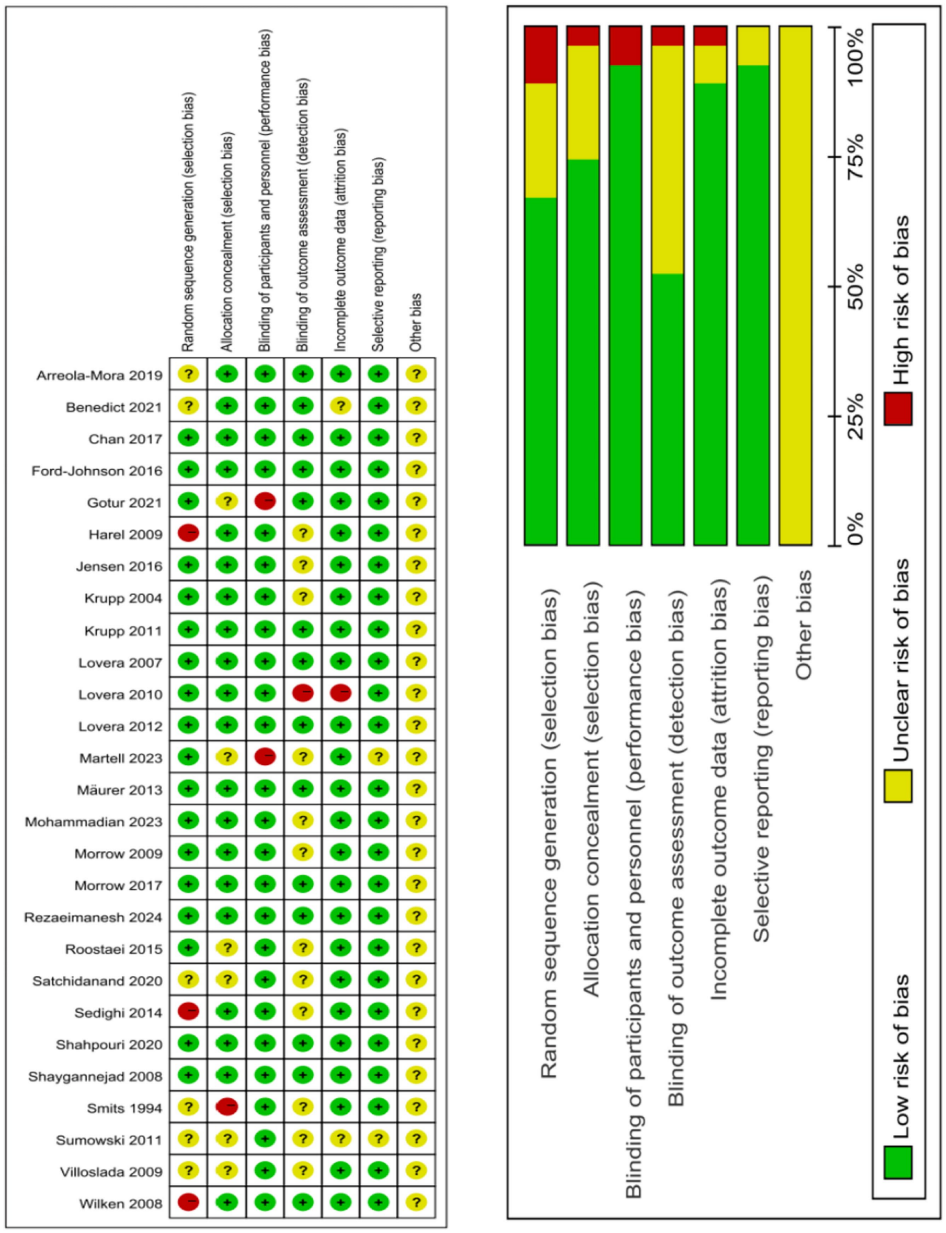
Risk of bias assessment for included studies.

Twenty five RCTs were included in our analysis, involving 1,726 participants. Among these, 940 patients had MS. The treatments investigated included rivastigmine, 4-aminopyridine (4-AP), donepezil, ginkgo biloba, memantine, modafinil, methylphenidate, L-amphetamine, simvastatin, fampridine-SR, dalfampridine, CorSeNs, atomoxetine, and lutein, with dosages ranging from 1.5 to 500 mg. The specific characteristics of the eligible studies are detailed in Table 2. The studies originated from various countries: 1 from India, 1 from the Netherlands, 11 from the United States, 5 from Iran, 1 from Spain, 1 from Germany, 1 from Denmark, 1 from Mexico, and 2 from the United Kingdom. All included studies clearly described the blinding methods. The primary risk of bias stemmed from deviations from the intended interventions. A risk of bias assessment for the selected studies is shown in Figure 2.

**Table 2:** Characteristics of studies included in the network meta-analysis.

Study	Year	Country	Sample size	Mean age	Gender (M/F)	Intervention	Outcomes
Gotur	2021	India	Rivastigmine:30 Placebo:30	Rivastigmine:35.9 Placebo:37.3	36/24	Rivastigmine:oral, 1.5 mg BD for 4 weeks	F1; F5
Smits	1994	The Netherlands	4-AP:10 Placebo:10	4-AMINOPYRIDINE:47.8 Placebo:46.8	5/15	4-AP: oral, 5 mg each per day, the dose was increased to four divided doses of 10 mg each during the second week.	F1; F7; F8
Krupp	2004	USA	Donepezil:35 Placebo:34	Donepezil:42.49 ± 9.27 Placebo:45.85 ± 7.65	22/47	Donepezil: oral, 5 mg per day, increasing to 10 mg per day at week 4.	F5; F7; F8
Lovera	2007	USA	*Ginkgo biloba*:21 Placebo:22	*Ginkgo biloba*:47.8 Placebo:50.2	12/27	*Ginkgo biloba*:oral, 120 mg Twice a day for 12 weeks.	F5; F7; F8
Shaygannejad	2008	Iran	Rivastigmine:30 Placebo:30	Rivastigmine:33.4 Placebo:31.6	27/33	Rivastigmine: oral, 1.5 mg once daily increment over 4 weeks to 3 mg twice daily for 12 weeks.	F1; F5
Villoslada	2009	Spain	Memantine:30 Placebo:30			Memantine: oral, 30 mg daily, starting with a titration dose of 10 mg per day and increasing by 10 mg each week	F5
Wilken	2008	USA	Modafinil + IM IFNβ-1a:23 IM IFNβ-1a:26	Modafinil + IM IFNβ-1a:48.2 IM IFNβ-1a:45.4		Modafinil + IM IFNβ-1a: titrated onto modafinil: modafinil 100 mg for each of the first 3 days, then 200 mg/day for the remainder of the 4-month study.	F5
Harel	2009	Israel	Methylphenidate:14 Placebo:12	Methylphenidate: 34.6 ± 10.2 Placebo:40.1 ± 10.5	6/20	Methylphenidate: oral, 10 mg	F7
Morrow	2009	USA	L-amphetamine:108 Placebo:43	L-amphetamine:47.8 ± 8.47 Placebo:50.4 ± 7.36	36/115	L-amphetamine: oral, 5 mg. After 7 days, grew to 15 mg, then to 30 mg after another 7 days. Maintained for 14 days.	F1; F5; F7; F8; F13
Lovera	2010	USA	Memantine:54 Placebo:60	Memantine:50.5 ± 8.2 Placebo:50.4 ± 7.7	19/95	Memantine: oral, two 5 mg capsules in the morning and evening	F5; F7; F8; F13
Krupp	2011	USA	Donepezil:61 Placebo:59	Donepezil:46.2 ± 7.5 Placebo:47.3 ± 8. 9	27/93	Donepezil: oral, 5 mg daily, and 10 mg daily at week 4	F5; F8
Sumowski	2011	USA	l-amphetamine:108 Placebo:49	l-amphetamine:48.5 ± 8.5 Placebo:48.5 ± 8.5	31/105	l-amphetamine: oral, 5 mg, which rose to 15 mg after 7 days, and 30 mg after another seven days. This 30 mg dose was kept for 14 days.	F1; F13
Lovera	2012	USA	*Ginkgo biloba*:61 Placebo:59	*Ginkgo biloba*:51.3 ± 8.6 Placebo:53 ± 9.5	54/66	*Ginkgo biloba*: oral, 120 mg twice a day for 12 weeks	F5; F7; F13
Mäurer	2013	Germany	Rivastigmine:43 Placebo:38	Rivastigmine:44.6 ± 9.4 Placebo:44.0 ± 7.3	38/43	Rivastigmine: patches of 5 cm^2^ (4.6 mg/day) for four weeks. Well tolerated, patients then received patches of 10 cm^2^ (9.5 mg/day) during the double-blind maintenance period for 12 weeks	F5; F7; F8
Roostaei	2015	Iran	Melatonin:13 Placebo:12	Melatonin:33.3 ± 7.6 Placebo:34.5 ± 8.2	4/21	Melatonin:oral, 3 mg, for 12 months	F7
Ford-Johnson	2016	USA	Modafinil:7 Placebo:9	Modafinil:41.67 ± 7.59 Placebo:43.43 ± 8.52	3/13	Modafinil:oral, 200 mg for 2 weeks	F1; F8; F13
Jensen	2016	Denmark	Modafinil:17 Placebo:20	Modafinil:50.8 ± 6.5 Placebo:48.4 ± 6.4	16/27	Modafinil:oral, 10 mg BID treatment.	F8
Chan	2017	UK	simvastatin:70 Placebo:70	Simvastatin:51.5 ± 7.0 Placebo:51.1 ± 6.8	68/68	Simvastatin:oral, 80 mg daily for 24 months	F1; F7
Morrow	2017	UK	Fampridine-SR:29 Placebo:31	Fampridine-SR:46.2 ± 10.7 Placebo:46.7 ± 9.6	14/46	Fampridine-SR:oral, 10 mg BID for 4 weeks	F7
Arreola-Mora	2019	México	4-AP:11 Placebo:10	4-AP:39.5 ± 8 Placebo:39.3 ± 9.8	8/13	4-AP:oral, 10 mg, three times a day with 6 h between every dose (e.g., 8 a.m. – 2 pm – 8 pm).	F1; F5; F7; F8
Satchidanand	2020	USA	dalfampridine:45 Placebo:16	dalfampridine:47.6 ± 10.2 Placebo:53.0 ± 7.5	13/48	dalfampridine:oral, 10 mg twice daily, 12-week period.	F1; F7; F8; F13
Shahpouri	2020	Iran	Donepezil:52 Placebo:51	Donepezil:31.92 ± 5.89 Placebo:0.66 ± 5.43	29/71	Donepezil:oral, daily 10 mg for 3 months	F1; F5
Rezaeimanesh	2024	Iran	Cor@SeNs:30 Placebo:30	Cor@SeNs group:41 ± 12.0 Placebo:35.0 ± 8.1	17/43	Cor@SeNs group:oral, 500 mg once daily, with a one-hour gap after a meal, twelve weeks	F1; F8; F13
Mohammadian	2023	Iran	atomoxetine:26 Placebo:26	atomoxetine:37.7 ± 8.5 Placebo:37.8 ± 7.6	37/15	atomoxetine:oral, 40 mg daily for 2 weeks and then 40 mg twice a day for 2.5 months	F1; F5; F8; F13
Martell	2023	USA	lutein:12 Placebo:9	Treatment:51.6 ± 10.0 Placebo:52.8 ± 8.1	2/19	Treatment: oral, comprised 20 mg/d, daily with a meal for 4 mo.	F8

F1: logical memory; F5:adverse events; F7: paced auditory serial addition test; F8: symbol digit modalities test; F13: CVLT-II long delay free (words) (CVLT).

### 3.2 Memory

**Figure 3:**
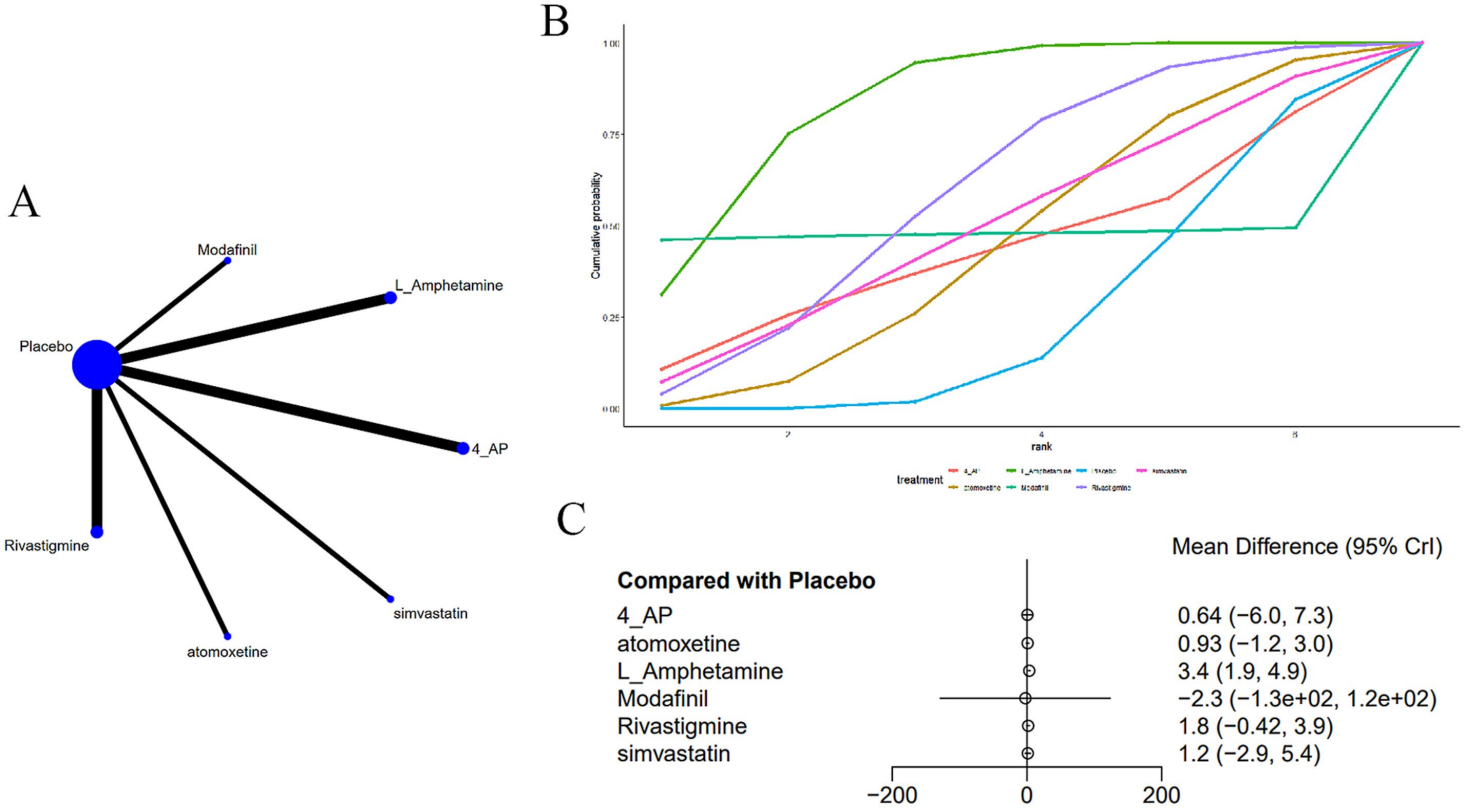
**(A)** Network plot; **(B)** area under the cumulative; **(C)** forest plot.

Nine studies (14, 17, 20, 24, 28, 30, 32, 35, 37) addressed memory (Figure 3). The network plot (Figure 3A) reveals that, in contrast to placebo, L-amphetamine [MD = 3.38, 95% CI (1.9, 4.88)] significantly improves memory performance in CD induced by MS (Figure 3C). There were no significant differences observed across various interventions (Supplementary Table S1). The cumulative area under the curve (AUC) analysis indicated that L-amphetamine had the highest AUC (83.3%), followed by rivastigmine (58.3%), simvastatin (48.9%), and placebo, which had the lowest AUC (24.5%) (Figure 3B).

### 3.3 PASAT

**Figure 4:**
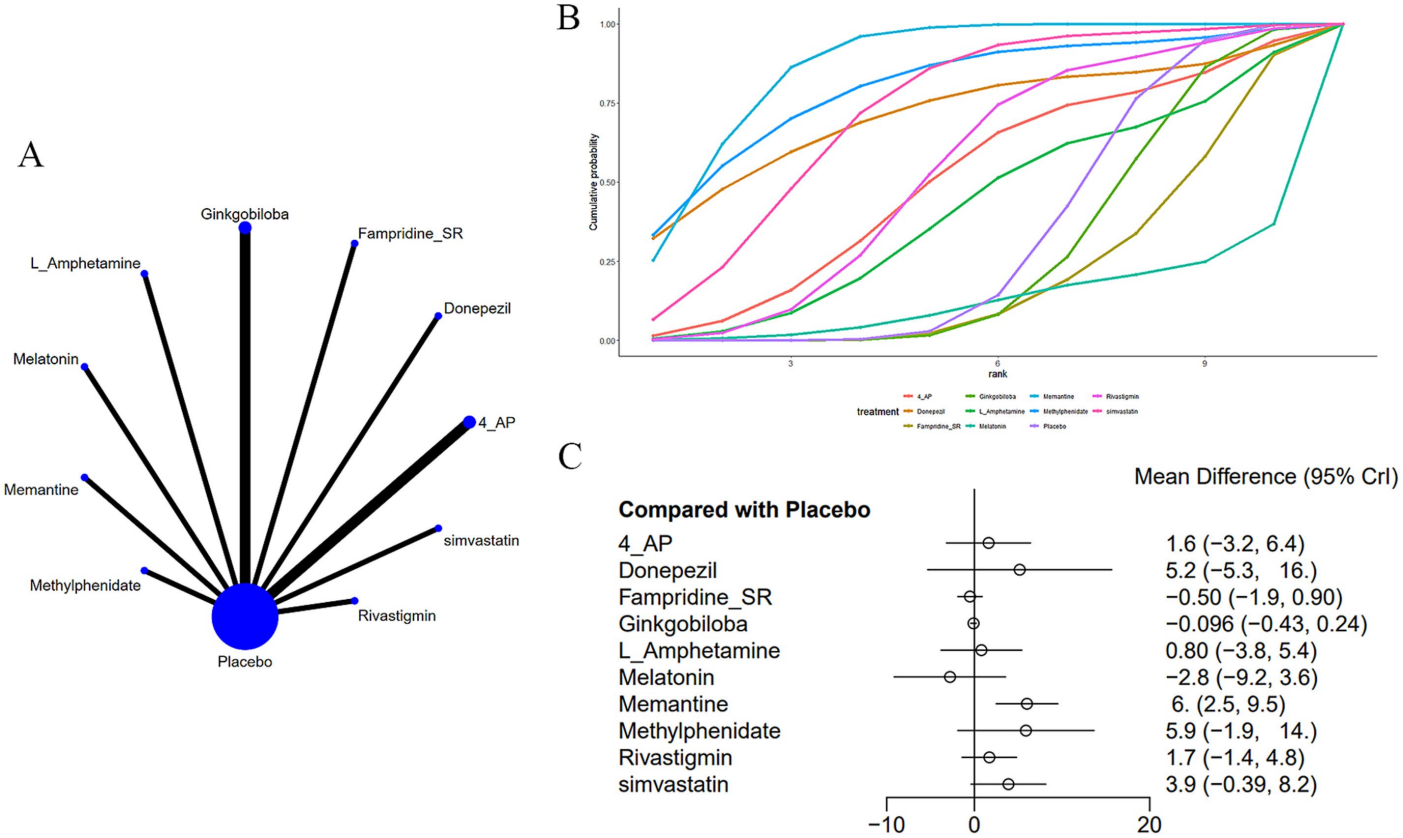
**(A)** Network plot; **(B)** area under the cumulative probability curve; **(C)** forest plot.

Twelve studies (14–16, 19, 20, 22, 25–27, 30–32) evaluated the PASAT (Figure 4). The network plot (Figure 4A) demonstrates that, as contrasted with placebo, memantine [MD = 6.0, 95% CI (2.49, 9.53)] significantly enhances performance on the PASAT in patients with MS-induced CD (Figure 4C). In comparison to memantine, fampridine_SR [MD = −6.49, 95% CI (−10.28, −2.72)], ginkgo biloba [MD = −6.1, 95% CI (−9.63, −2.57)], and melatonin [MD = −8.78, 95% CI (−16.09, −1.5)] all showed inferior effects. Memantine demonstrated superior efficacy over fampridine_SR, ginkgo biloba, and melatonin (Supplementary Table S2). The cumulative AUC analysis revealed that L-amphetamine achieved the highest AUC (86.8%), followed by methylphenidate (79.8%), simvastatin (72.0%), with melatonin showing the lowest AUC (12.7%) (Figure 4B).

### 3.4 SDMT

**Figure 5:**
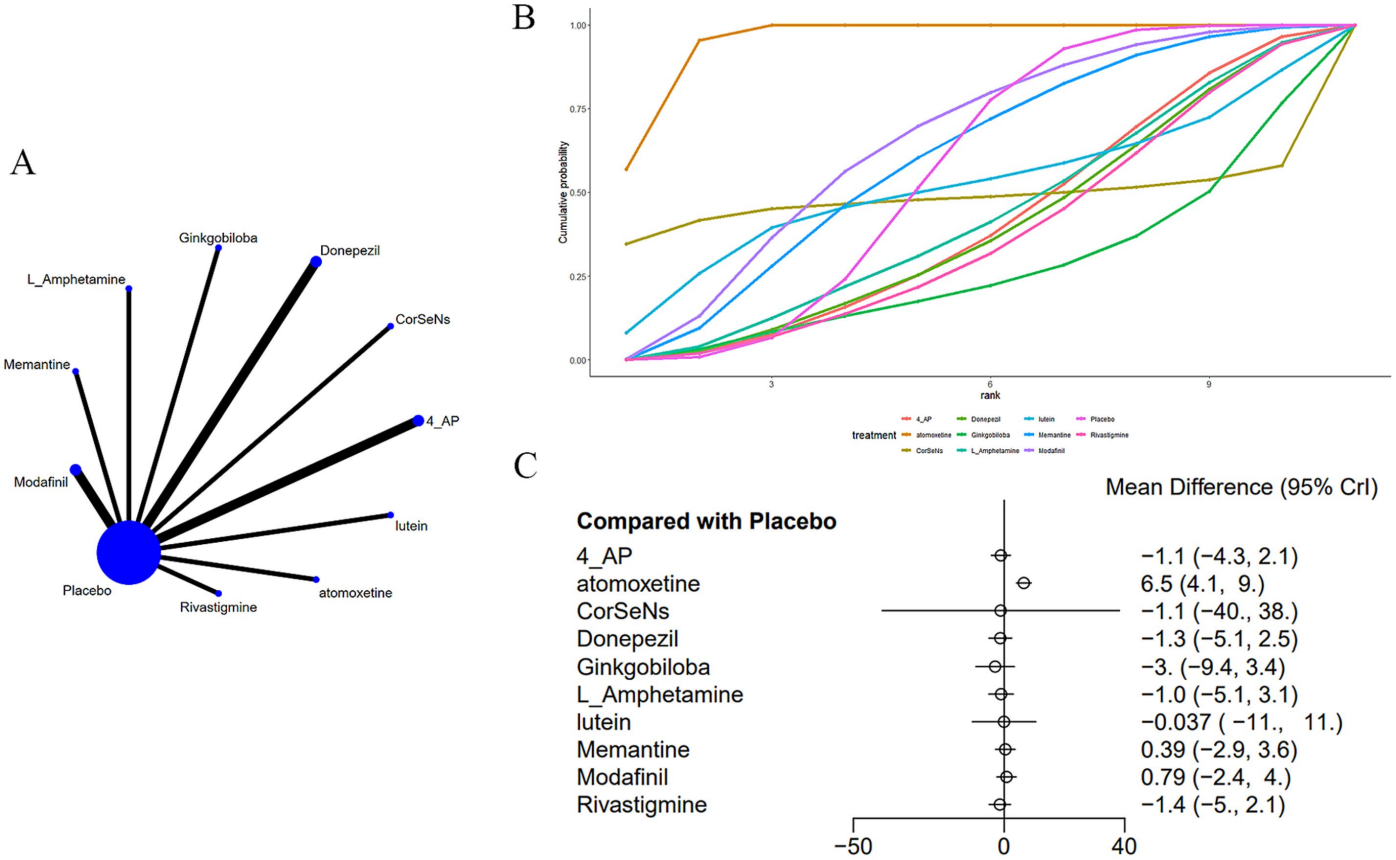
**(A)** Network plot; **(B)** area under the cumulative probability curve; **(C)** forest plot.

Thirteen studies (14–16, 20, 22, 23, 26, 28, 29, 32, 36–38) reported on SDMT, as shown in Figure 5. The network diagram (Figure 5A) indicated that, compared to a placebo, atomoxetine [MD: 6.53, 95% CI (4.07, 8.99)] may improve SDMT in CD induced by MS. Furthermore, the effect of atomoxetine was superior to that of donepezil [MD: 7.8, 95% CI (3.26, 12.29)], ginkgo biloba [MD: 9.51, 95% CI (2.67, 16.38)], L-amphetamine [MD: 7.54, 95% CI (2.77, 12.31)], modafinil [MD: 5.73, 95% CI (1.7, 9.79)], and rivastigmine [MD: 7.93, 95% CI (3.62, 12.26)] (Supplementary Table S3). The AUC (Figure 5B) showed that atomoxetine at 40 mg had the greatest probability (95.2%), followed by modafinil at 10 mg (63.9%), while ginkgo biloba at 120 mg had the lowest probability (25.7%).

### 3.5 CVLT

**Figure 6:**
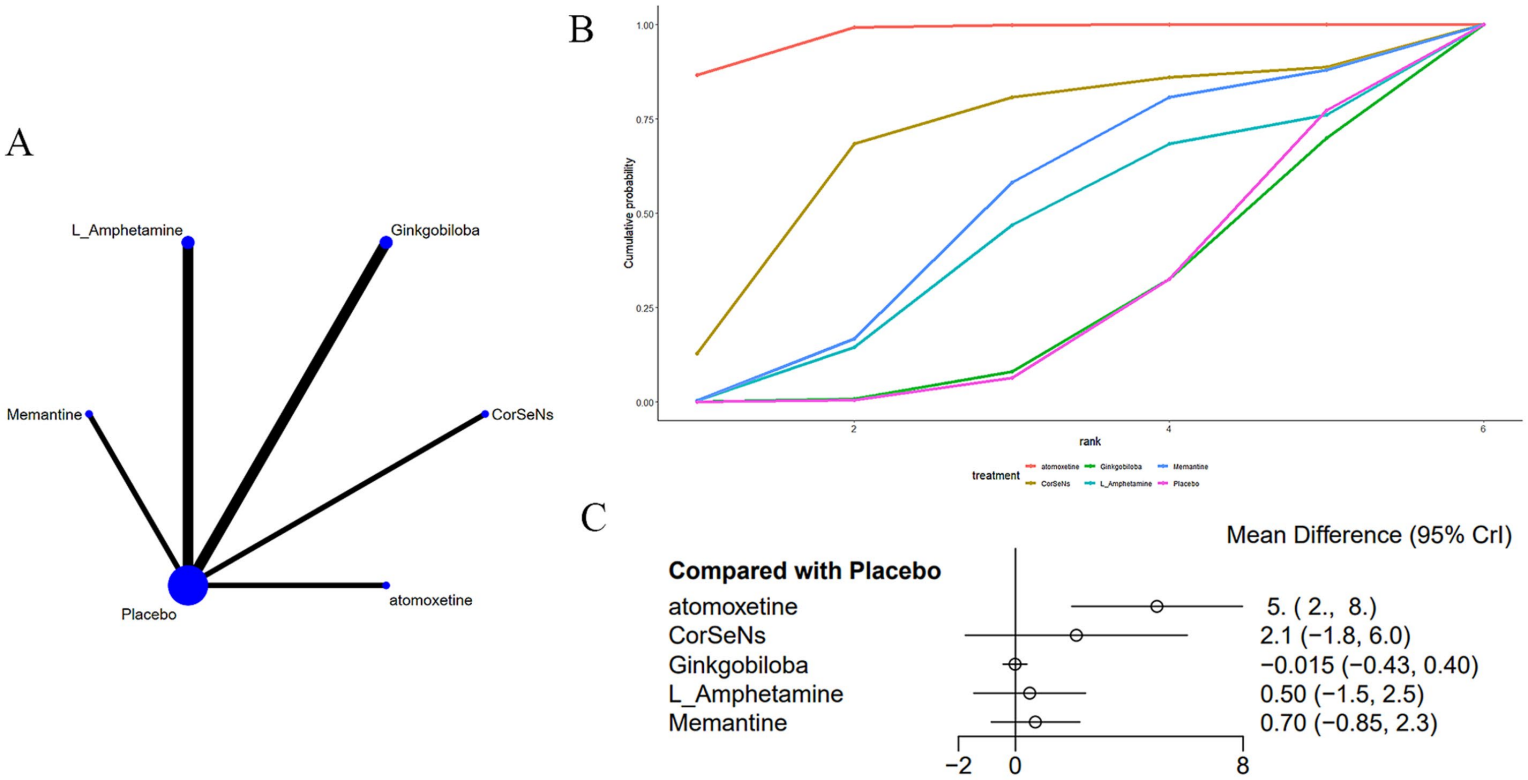
**(A)** Network plot; **(B)** area under the cumulative probability curve; **(C)** forest plot.

Seven studies (16, 20, 22, 24, 25, 37, 38) reported on the CVLT, as shown in Figure 6. The network diagram (Figure 6A) demonstrated that, compared to a placebo, atomoxetine [MD: 4.97, 95% CI (1.98, 7.98)] may improve CD induced by MS, as assessed by the CVLT (Figure 6C). Additionally, the effect of atomoxetine was superior to that of ginkgo biloba [MD: 4.99, 95% CI (1.98, 7.98)], L-amphetamine [MD: 4.47, 95% CI (0.89, 8.07)], and memantine [MD: 4.27, 95% CI (0.89, 7.66)] (Supplementary Table S4). The AUC (Figure 6B) indicated that atomoxetine had the greatest probability (97.1%), followed by CorSeNs (67.3%), with ginkgo biloba having the lowest probability (22.3%).

### 3.6 Nausea

**Figure 7:**
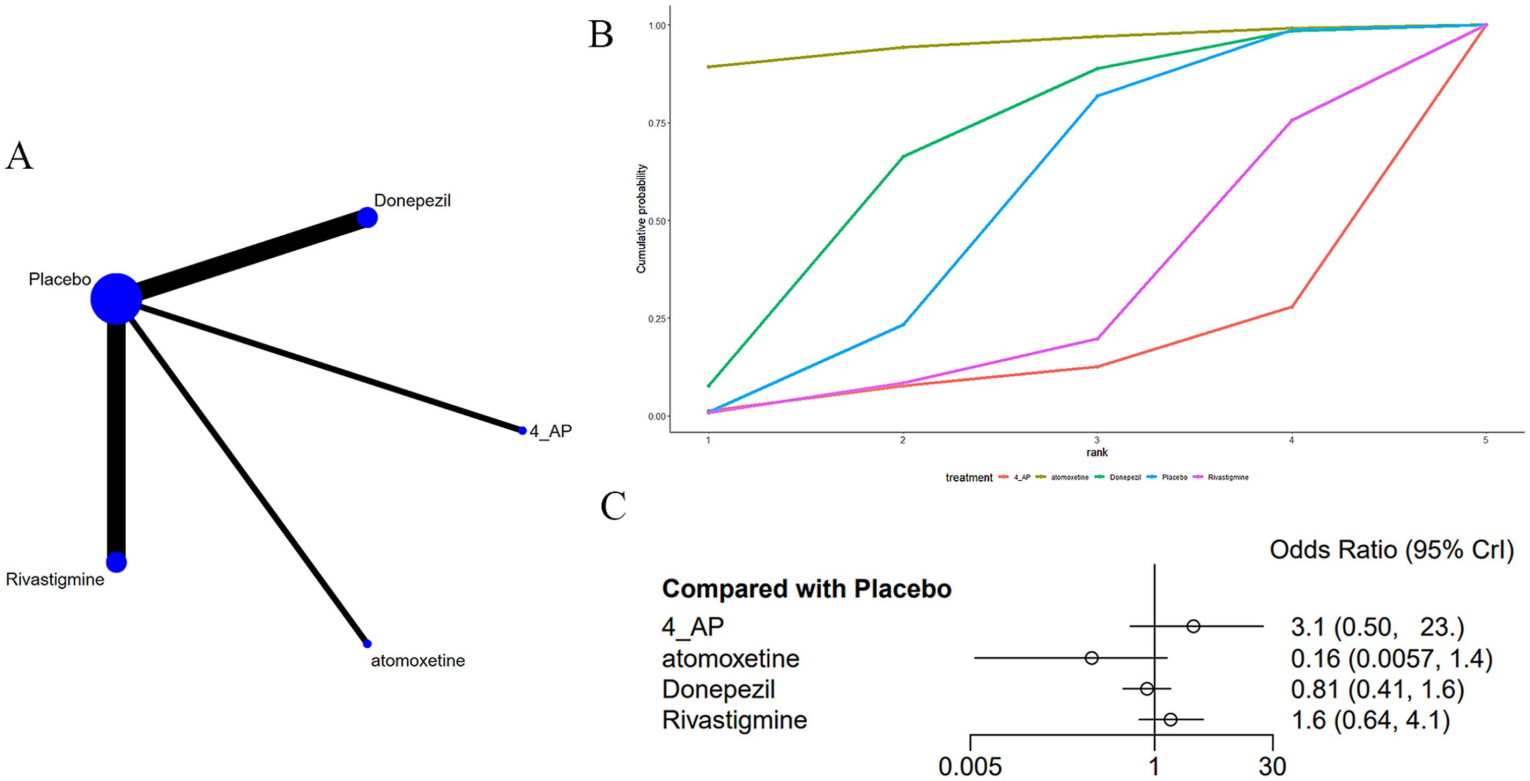
**(A)** Network plot; **(B)** area under the cumulative probability curve; **(C)** forest plot.

Eight studies (15, 17, 23, 26, 32, 34, 35, 37) reported adverse drug reactions, specifically nausea, associated with medications used to treat MS-induced CD, as shown in Figure 7. The network diagram (Figure 7A) suggested no notable variations across treatments when compared with a placebo (Figure 7C) (Supplementary Table S5). The AUC (Figure 7B) showed that atomoxetine had the greatest probability (95.0%), while 4-AP had the lowest (12.3%).

### 3.7 Insomnia

**Figure 8:**
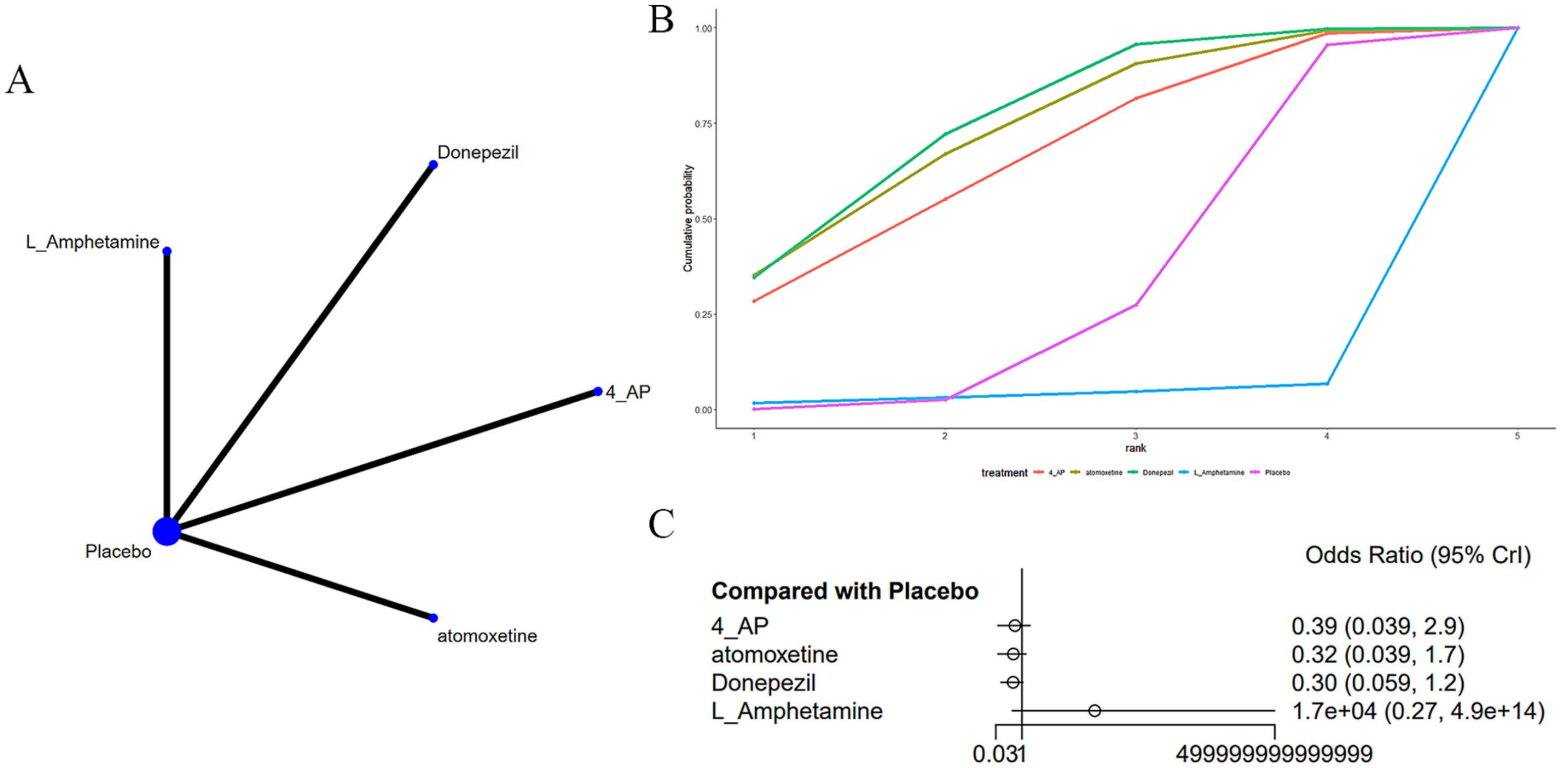
**(A)** Network plot; **(B)** area under the cumulative probability curve; **(C)** forest plot.

Four studies (20, 23, 32, 37) reported insomnia as an adverse drug reaction in MS-induced CD treatment, as shown in Figure 8. The network diagram (Figure 8A) did not indicate marked differences in various treatments in comparison to a placebo (Figure 8C) (Supplementary Table S6). According to AUC (Figure 8B), donepezil had the highest probability (75.6%), while L-amphetamine had the lowest (0.04%).

### 3.8 Dyspepsia

**Figure 9:**
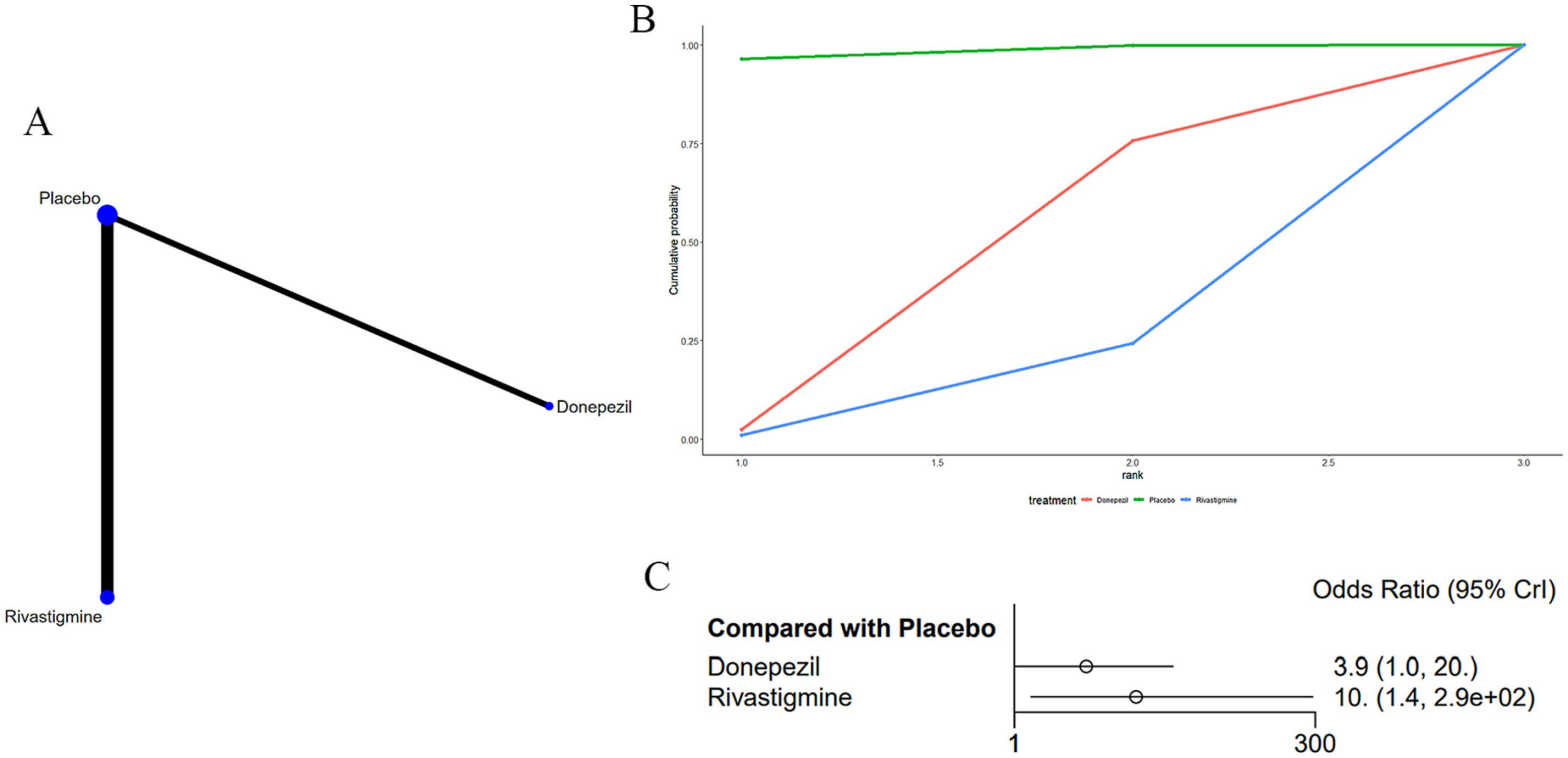
**(A)** Network plot; **(B)** area under the cumulative probability curve; **(C)** forest plot.

Three studies (15, 17, 35) reported dyspepsia as an adverse drug reaction associated with treatments for MS-induced CD, as displayed in Figure 9. The network diagram (Figure 9A) indicated that rivastigmine [OR: 0.1, 95% CI (0, 0.73)] was less likely to cause dyspepsia compared to a placebo (Supplementary Table S7).

According to AUC (Figure 9B), donepezil had the highest probability (39.1%), while rivastigmine had the lowest (12.7%).

### 3.9 Evaluation of publication bias

Funnel plots were used to evaluate the publication bias of memory, adverse reactions, Paced Auditory Serial Addition Test, Symbol Digit Modalities Test, and California Verbal Learning Test in patients with multiple sclerosis-induced cognitive dysfunction. According to the parallelism of the horizontal line in the funnel plot with the *x*-axis, there was no publication bias in the original studies (Supplementary Figures S1–S7).

## 4 Discussion

This is the first evaluation of the roles of various dosages and sorts of medications in memory, auditory rhythm-sequence addition tasks, SDMT, CVLT, and adverse outcomes in patients with CD induced by MS. The network meta-analysis conducted not only allows for the comparison of different drugs but also enables indirect comparisons to assess the overall therapeutic efficacy, marking the innovation of this study. The results show that, in terms of improving memory function in MS-induced CD, L-amphetamine was the most effective, followed by rivastigmine, with placebo performing the worst. Melatonin might enhance performance on the auditory rhythm-sequence addition task in MS-related cognitive impairment, while L-amphetamine yielded the highest AUC, with melatonin yielding the lowest. Atomoxetine potentially improved SDMT performance, with atomoxetine showing the largest AUC, and ginkgo biloba performing the worst. Additionally, atomoxetine might also improve performance on the CVLT. However, when considering adverse drug reactions in MS-related CD, rivastigmine was associated with a lower incidence of dyspepsia.

Research by Ke et al. (39) indicates that amphetamine has a role in dopamine (DA) synthesis, storage, release, and reuptake, acting as a stimulant and both cognitive and physical enhancer, with applications in treating various cognitive disorders, though it carries a high risk of abuse. A study by Jian-Min et al. (40) compared the influences of d-amphetamine and lisdexamfetamine on cognitive functions associated with the medial prefrontal cortex (mPFC) using tasks like the Y-maze spontaneous alternation and delayed non-match to sample tests, which assess spatial working memory and spatial recognition memory, respectively. Through microdialysis, the study assessed DA and its metabolites in the mPFC of freely moving rats, exploring the neurochemical profiles induced by varied pharmacokinetics of d-amphetamine and lisdexamfetamine.

In a 2:1 randomized, placebo-controlled, double-blind trial concerning 33 MS clinics in America, Morrow et al. (20) investigated the roles of 30 mg oral L-amphetamine over 29 days, including a dose-escalation phase. The results demonstrated that L-amphetamine sulfate was associated with improvements in learning and memory and was well tolerated. Nevertheless, as positive results were noted only in secondary outcome measures, further replication studies are required before L-amphetamine sulfate is advised for treating CD in MS.

It has been reported that clinically inappropriate doses of amphetamine, either too low or too high, can adversely affect DA levels and impair cognition, thus limiting its widespread use. Modifying the pharmacokinetics of amphetamine to enhance cognitive effects while reducing the potential for abuse presents a challenge. Another strategy is to alter its chemical structure by conjugating the active drug with another compound, like an amino acid, to develop a new prodrug.

Therefore, lisdexamfetamine may have a wider therapeutic window for cognitive improvement compared to d-amphetamine. While this study explored the differences between various amphetamine isomers, and certain data regarding L-amphetamine demonstrate statistical significance, their clinical relevance remains minimal and should therefore be interpreted with caution. More research is needed to further validate our conclusions regarding dosage and drug selection, which may provide additional treatment options for patients with MS-induced CD. L-amphetamine.

Memantine, a pharmacological agent classified as an N-methyl-D-aspartate (NMDA) receptor antagonist, exerts its effects by attenuating excessive NMDA receptor activation and counteracting elevated glutamate levels in the brain. Through this mechanism, it helps protect neurons against glutamate-induced excitotoxicity and holds potential for enhancing neural signal transmission.

Moreover, memantine has been shown to sustainably potentiate synaptic connections between simultaneously activated neurons. In randomized controlled trials conducted in patients with Alzheimer’s disease, memantine significantly slowed cognitive decline and loss of functional independence compared with placebo, while demonstrating favorable tolerability. Currently, memantine is among the available pharmacological options for dementia, primarily prescribed in moderate-to-severe Alzheimer’s disease. Nevertheless, its clinical value remains debated, as its therapeutic efficacy is limited and it does not halt the progression of neurodegenerative pathology (41).

Previous findings have suggested that memantine reduces neuronal damage, improves cerebral blood flow in both global and focal ischemia models, and enhances cognitive performance across various stages of dementia. Animal studies further indicate that memantine may ameliorate memory deficits and mitigate ischemia-induced brain injury (42).

Accumulated evidence demonstrates that cognitive impairment in multiple sclerosis (MS) represents a form of subcortical dementia, characterized primarily by deficits in memory, information processing speed, and executive function. Another proposed mechanism underlying MS-related cognitive decline is glutamate-mediated excitotoxicity, a hypothesis supported by *in vivo* models showing that elevated glutamatergic activity induces neuronal excitotoxicity and consequent cognitive deterioration.

At present, no established clinical guidelines exist for the management of cognitive impairment in MS. Recent investigations have indicated that DMTs can attenuate neurodegeneration and, subsequently, reduce the incidence of cognitive dysfunction.

The rationale for considering memantine as a potential prophylactic therapy for cognitive impairment in MS is derived from the pathophysiological hypothesis that excessive glutamatergic activity within CNS plaques may contribute to the development of cognitive deficits.

Future studies are warranted to further evaluate the potential benefits of memantine in MS-related cognitive impairment. Such investigations should consider different MS clinical subtypes, the effects of memantine in combination with DMTs, longer treatment durations, and the implementation of more standardized and sensitive outcome measures (43).

Atomoxetine, an effective selective norepinephrine reuptake inhibitor, enhances the availability of synaptic norepinephrine within the CNS. It can ameliorate higher cognitive functions in patients with Attention Deficit Hyperactivity Disorder (ADHD). Additionally, atomoxetine specifically increases extracellular DA levels in mPFC, suggesting that the therapeutic mechanisms of atomoxetine may extend beyond previous understandings. However, the neuropharmacological aspects of this drug still require further investigation (44).

Levey et al.’s (45) biomarker-driven, double-blind, crossover trial of atomoxetine demonstrated that, compared to placebo, atomoxetine significantly correlated with a decrease in cerebrospinal fluid (CSF) levels of Tau and pTau181, but exhibited no significant correlation with changes in amyloid-β42. Atomoxetine treatment also markedly changed the abundance of a protein panel in the CSF associated with brain pathophysiology, including synaptic, metabolic, glial immune proteins, and inflammation-related markers like CDCP1, CD244, TWEAK, and osteoprotegerin. This treatment was also associated with a significant increase in brain-derived neurotrophic factor (BDNF) in plasma and a reduction in triglycerides. Resting-state functional MRI revealed a significant increase in network connectivity between the insula and hippocampus due to the effects of atomoxetine. Fluorodeoxyglucose (FDG)-PET imaging displayed elevated uptake in the hippocampus, parahippocampal gyrus, middle temporal gyrus, inferior temporal gyrus, as well as fusiform gyrus, with a carryover effect observed at 6 months post-treatment.

The study by Park et al. (46) reviewed the information of 106 patients suffering post-stroke aphasia and cognitive impairment, and found that atomoxetine notably ameliorated cognitive and language functions in the post-stroke aphasia population. This was the first large-scale evaluation of the efficacy of atomoxetine in treating post-stroke cognitive impairment and aphasia.

According to the aforementioned article, atomoxetine has shown beneficial effects on cognitive improvement in both limited animal and human investigations. Nejad et al. (37) conducted a parallel, randomized clinical trial. Following the inclusion and exclusion criteria, 52 participants were enrolled and randomized into two groups of 26 each. The experimental cohort received atomoxetine treatment, while the control cohort was given a placebo. The Minimal Assessment of Cognitive Function in MS (MACFIMS) was performed at baseline and 3 months later. Cognitive changes post-treatment were evaluated using the CVLT, CVLT-Delayed, the Simple Visual–Spatial Memory Test, as well as SDMT. The results revealed a significant amelioration in cognitive levels after atomoxetine treatment in comparison to the placebo cohort (*p* < 0.05). Therefore, atomoxetine ameliorates cognitive functions in MS patients.

In previous studies, the pharmacological agents most frequently reported can be categorized into five classes: acetylcholinesterase inhibitors, central nervous system stimulants, fampridine, herbal preparations, and other medications. The overall heterogeneity across studies was moderate. No significant effects of treatment on cognitive function were observed for any of the assessed tasks (*p* < 0.05). However, in subgroup analyses, relevant findings demonstrated that fampridine treatment was associated with a significant improvement in SDMT performance (SMD = 0.283, 95% CI: 0.015–0.550, *p* = 0.039, *I*^2^ = 11.7%). The meta-analysis highlights that, to date, the proposed pharmacological treatments confer no clear benefit in alleviating MS-related cognitive impairment.

The included studies are subject to several limitations. First, the reported outcomes may have been confounded by publication bias. Second, only double-blind randomized controlled trials were considered as the most reliable sources of evidence, which excluded observational studies and may have influenced the overall conclusions. Third, the classification of drugs was based on their primary mechanisms of action; however, heterogeneity existed among medications within the same subgroup. Fourth, unreported outcomes may have been present in the included studies, which could substantially affect the results. Future investigations should focus on identifying effective pharmacological interventions for MS-induced cognitive impairment (47).

In summary, further research is required to explore the potential of atomoxetine in treating CD induced by MS, as it may be a prospective therapeutic option to slow the progression of MS-related cognitive decline. Although this study explored variations in drug types, dosages, and adverse reactions, our findings suggest that the differences among the top-ranking interventions in the league table were not significant. More research is required to substantiate our conclusions regarding dosage selection. Nonetheless, this study offers a potential treatment option for patients suffering from MS-induced CD.

### 4.1 Advantages and limitations

Our study has strengths and limitations. First, this research focused on identifying the best drug treatment for CD induced by MS, an area that has not been elucidated in the past. Second, only RCTs were selected and deemed as the “gold standard” in clinical research. Nevertheless, both our study and the eligible primary studies have certain limitations. Heterogeneity among the primary studies is inevitable (e.g., differences in male and female participants, origin, drug dosage selection, and sample size), which may influence the scientific validity of the network meta-analysis to a certain extent.

In the consensus published in 2022, multiple associations defined clinically meaningful change (CMC) variables for monitoring disease progression. For instance, a 20% decline in cognitive test scores (e.g., SDMT) or deterioration of at least 20% in two or more cognitive subtests is considered a clinically meaningful indicator of cognitive progression. The consensus recommends that these assessments be interpreted in conjunction with imaging evidence of brain or spinal cord atrophy (e.g., sustained or confirmed atrophy) to enhance specificity. Recommendations from various MS associations consistently emphasize that “clinically meaningful change” should be employed to evaluate disease activity, treatment response, and progression, encompassing objective measures of disability, cognitive function, and motor performance (e.g., changes in EDSS, SDMT score reduction, or increased walking test time). These recommendations are grounded in standardized tools and confirmatory assessments (e.g., confirmation within 3–6 months) to guide therapeutic decisions, such as DMT switching, and overall patient management.

Current network meta-analyses evaluate treatment efficacy based on statistical parameters (e.g., demonstrating higher SDMT and CVLT scores with tomoxetine compared with other interventions) but do not address whether these observed differences meet or exceed thresholds for clinical meaningfulness. This limitation may restrict the interpretability of study findings in real-world clinical practice, as statistically significant changes in cognitive test scores do not always correspond to functionally meaningful improvements for MS patients with cognitive impairment. Incorporating “clinically meaningful change” criteria would enhance the translational value of such studies, align them with the consensus recommendations of MS associations, and strengthen the relevance of conclusions for informing clinical decision-making.

Despite these limitations, it is hoped that future original research will expand upon our findings, providing more reliable clinical evidence-based recommendations.

## 5 Conclusion

Based on current research, among the medications for improving MS-induced CD, L-amphetamine is most effective in enhancing memory performance, while memantine best improves the PASAT scores. Atomoxetine was found to be most effective in enhancing SDMT scores and the CVLT. Regarding adverse reactions, rivastigmine is less likely to cause indigestion. In view of the limitations and heterogeneity inherent in the included studies, the conclusions should be interpreted with caution.

## Data Availability

The original contributions presented in the study are included in the article/Supplementary material, further inquiries can be directed to the corresponding author.

## Supplementary material

SUPPLEMENTARY FIGURE S1 Funnel plot for memory.

SUPPLEMENTARY FIGURE S2 Funnel plot for paced auditory serial addition test.

SUPPLEMENTARY FIGURE S3 Funnel plot for symbol digit modalities test.

SUPPLEMENTARY FIGURE S4 Funnel plot for California Verbal Learning Test.

SUPPLEMENTARY FIGURE S5 Funnel plot for nausea.

SUPPLEMENTARY FIGURE S6 Funnel plot for insomnia.

SUPPLEMENTARY FIGURE S7 Funnel plot for indigestion.
