# Supplementary figures and images for: Pharmacological treatment options for cognitive dysfunction induced by multiple sclerosis: a network meta-analysis
Source: Front Neurol. 2025 Oct 7;16:1649429. doi: 10.3389/fneur.2025.1649429 (PMC12537379; doi:10.3389/fneur.2025.1649429)

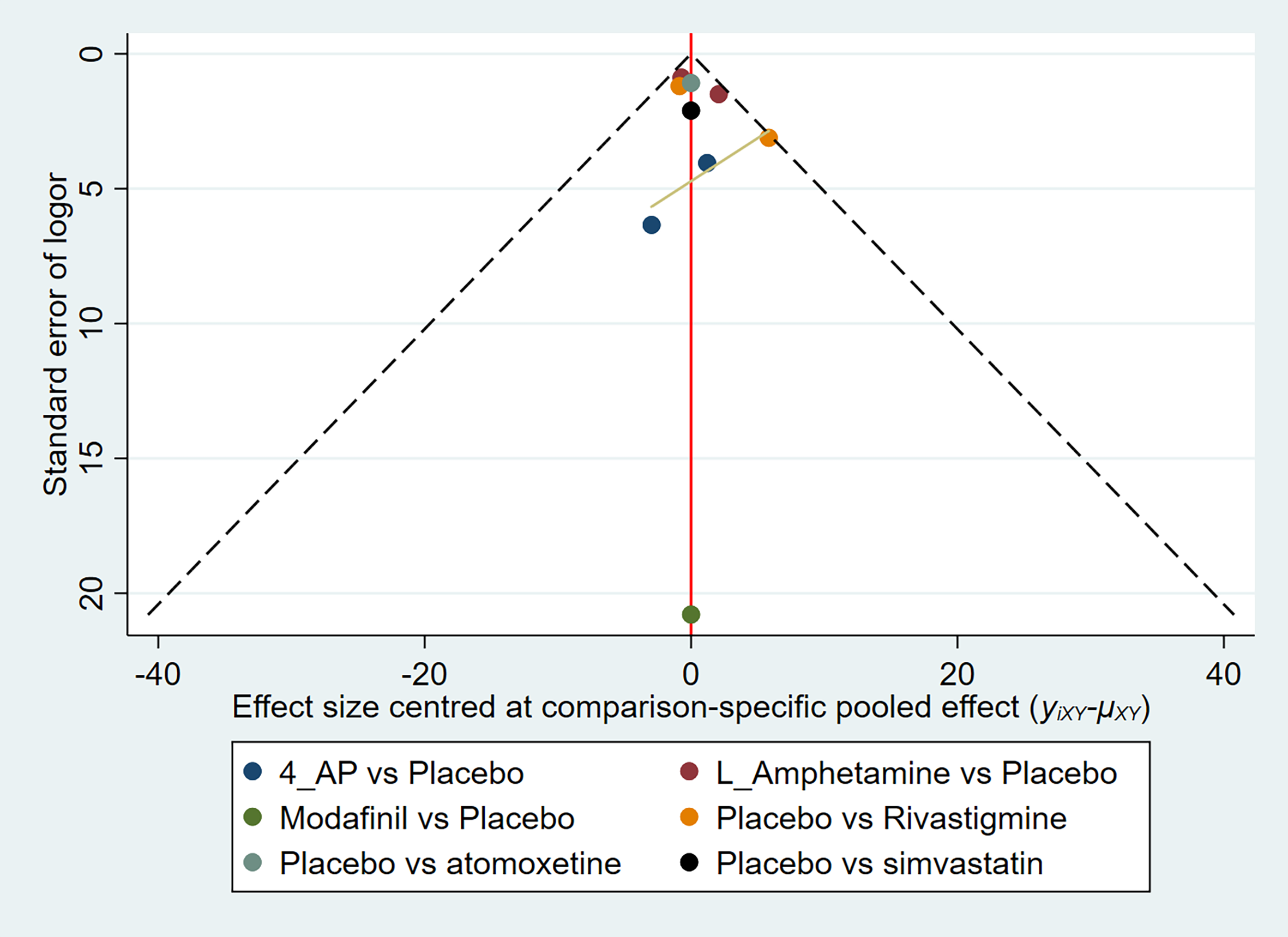

Supplement: SUPPLEMENTARY FIGURE S1 — Funnel plot for memory. [file Image_1.TIF]

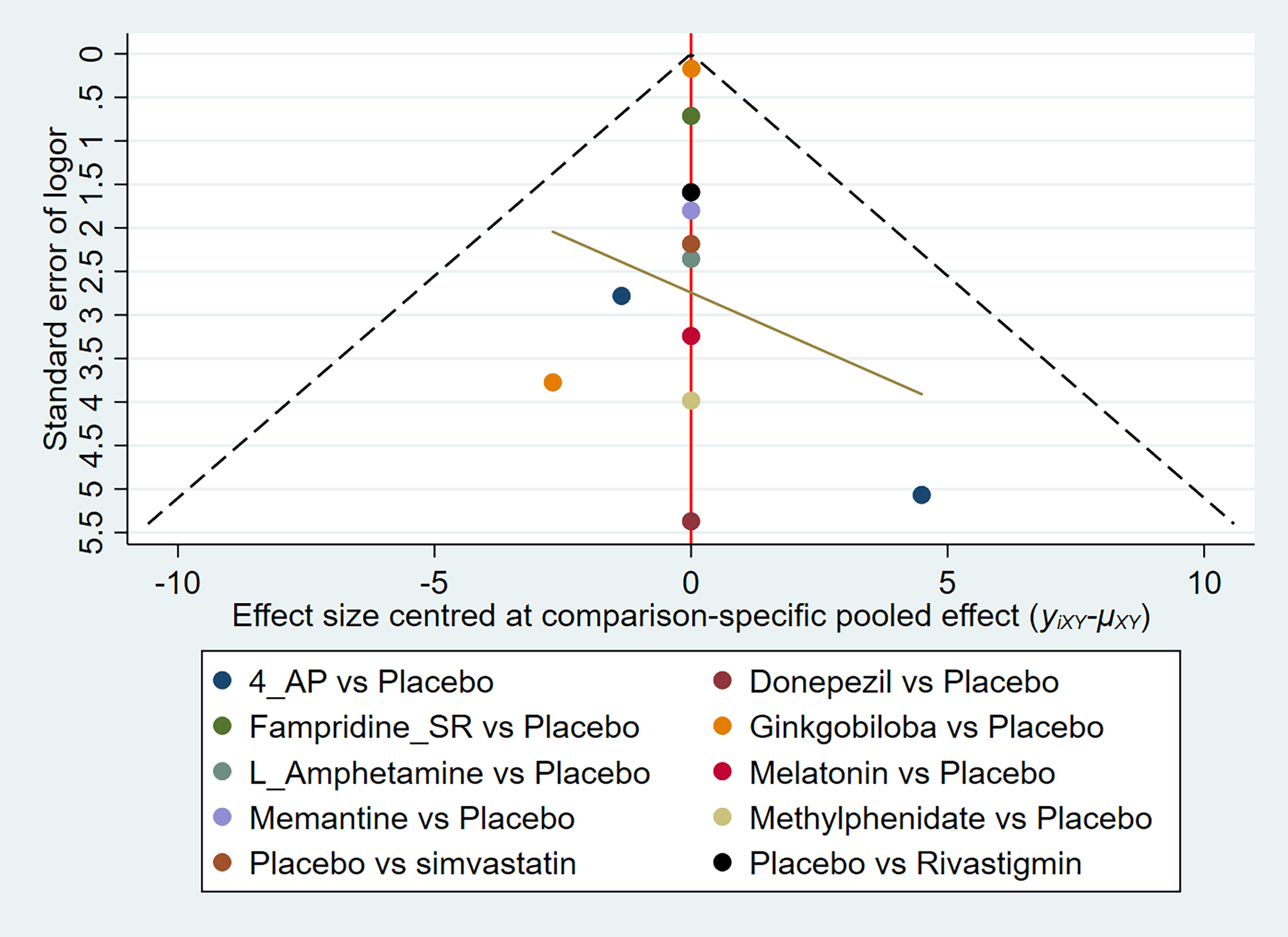

Supplement: SUPPLEMENTARY FIGURE S2 — Funnel plot for paced auditory serial addition test. [file Image_2.TIF]

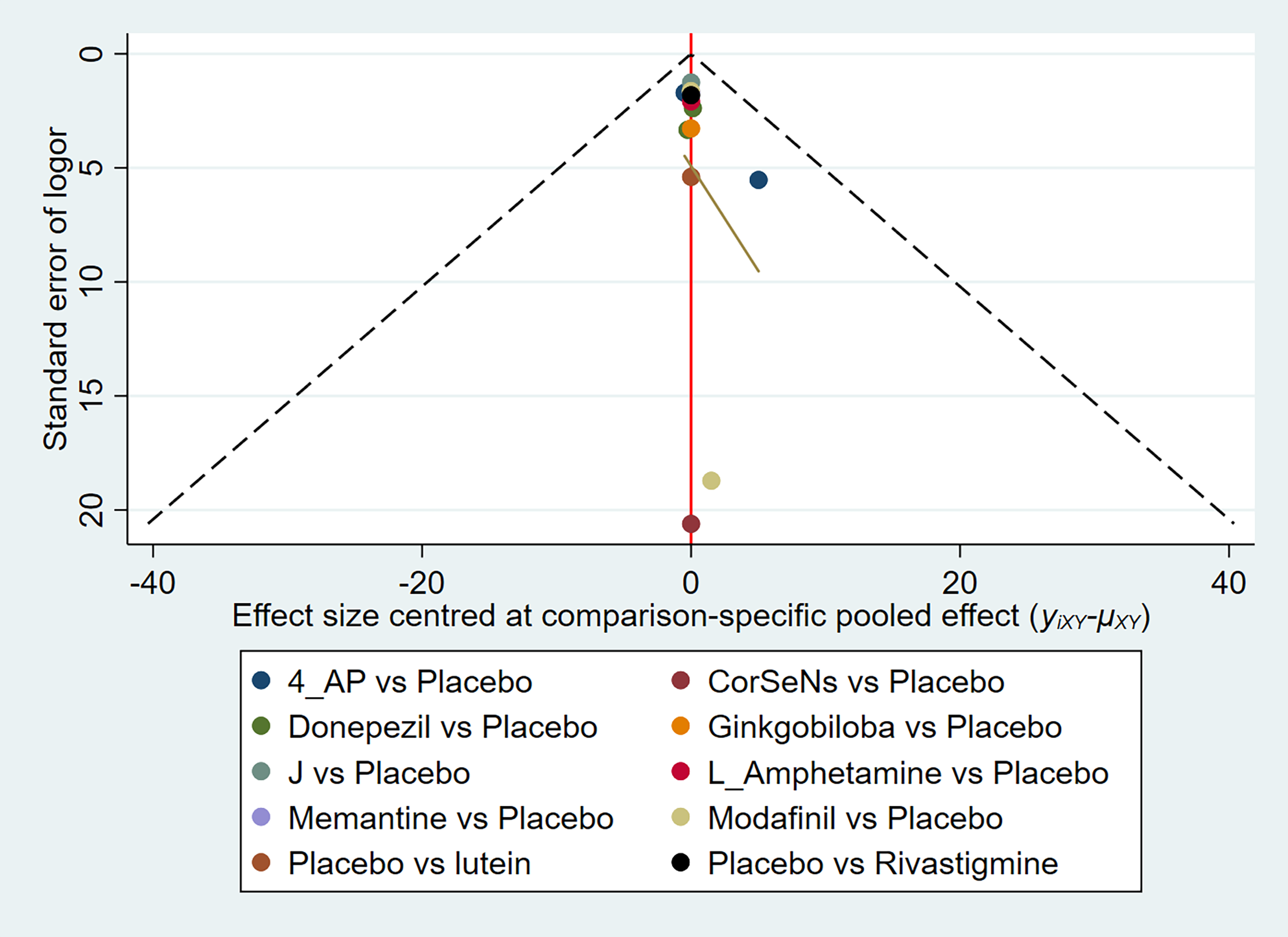

Supplement: SUPPLEMENTARY FIGURE S3 — Funnel plot for symbol digit modalities test. [file Image_3.TIF]

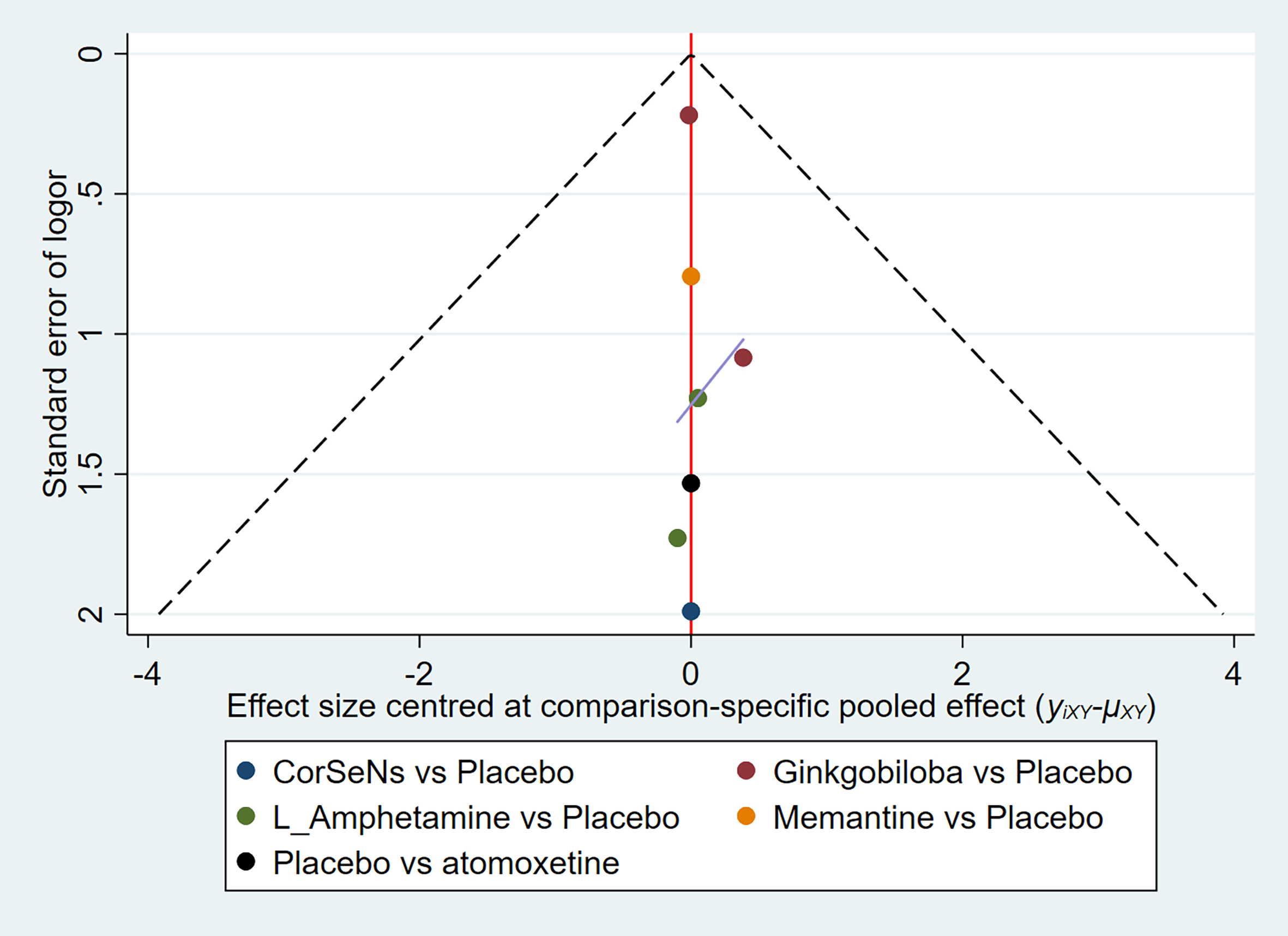

Supplement: SUPPLEMENTARY FIGURE S4 — Funnel plot for California Verbal Learning Test. [file Image_4.TIF]

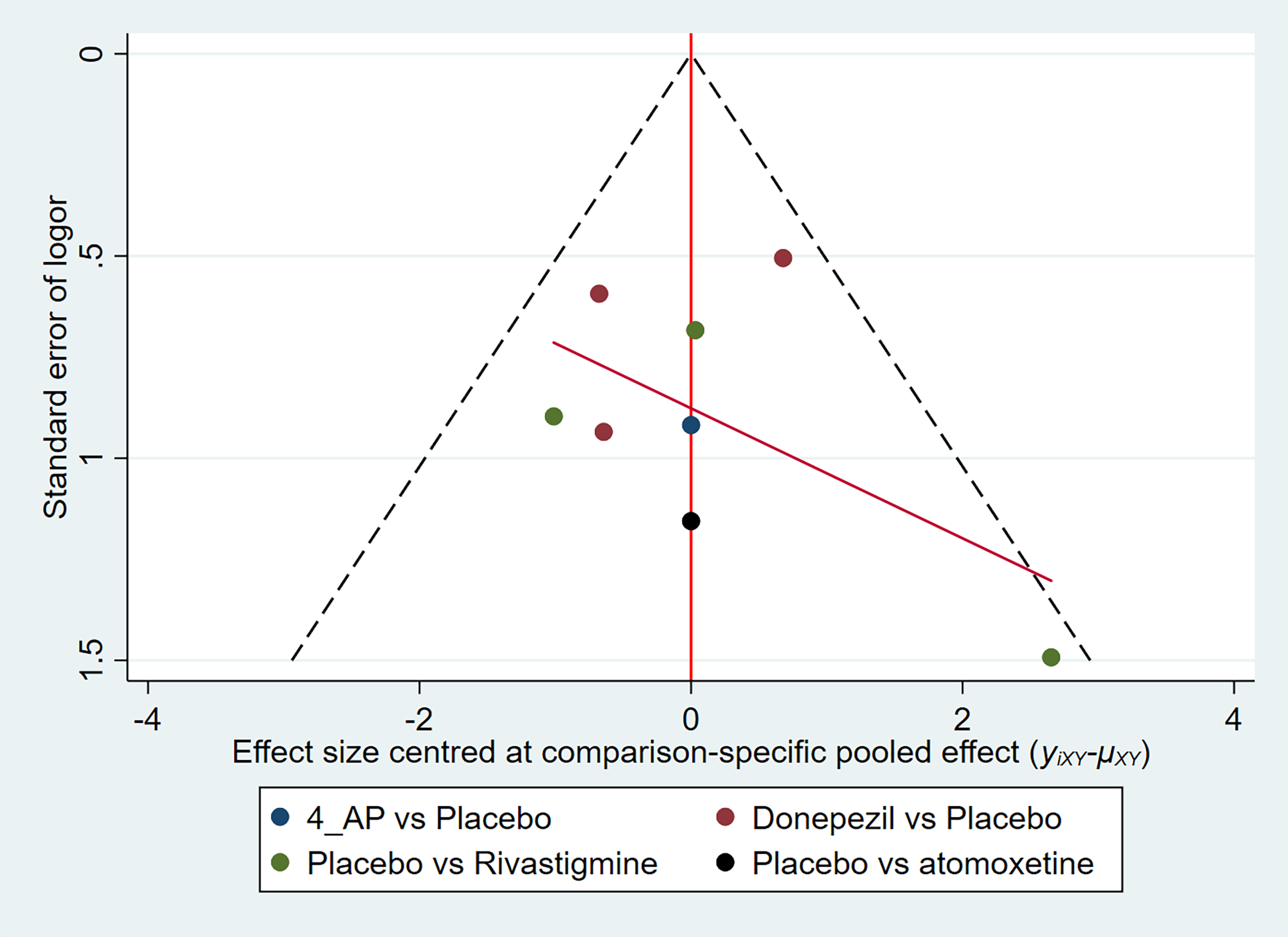

Supplement: SUPPLEMENTARY FIGURE S5 — Funnel plot for nausea. [file Image_5.TIF]

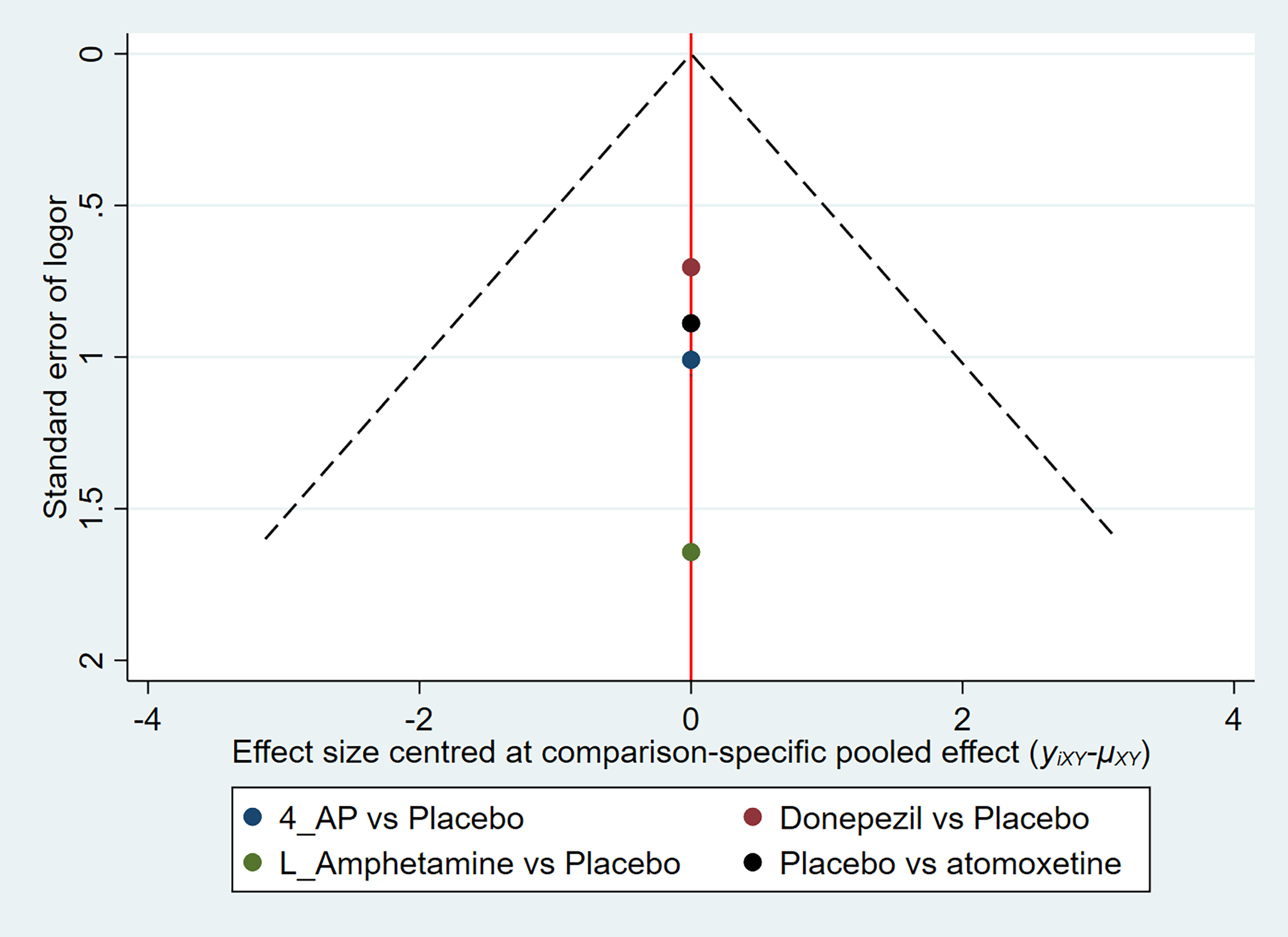

Supplement: SUPPLEMENTARY FIGURE S6 — Funnel plot for insomnia. [file Image_6.TIF]

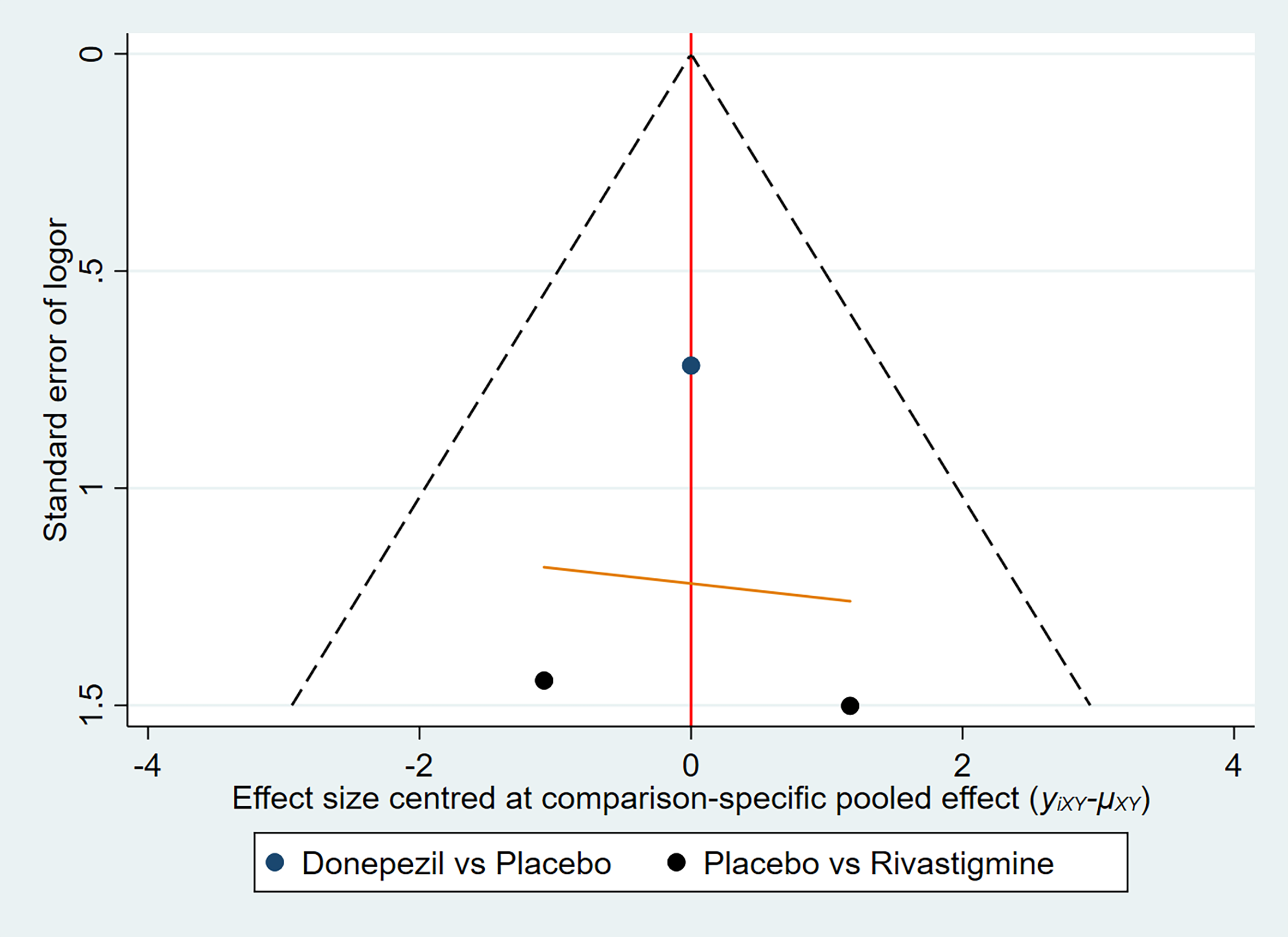

Supplement: SUPPLEMENTARY FIGURE S7 — Funnel plot for indigestion. [file Image_7.TIF]
