# Supplementary material for: Pharmacological treatment options for cognitive dysfunction induced by multiple sclerosis: a network meta-analysis
Source: Front Neurol. 2025 Oct 7;16:1649429. doi: 10.3389/fneur.2025.1649429 (PMC12537379; doi:10.3389/fneur.2025.1649429)
Supplement: Supplementary file 8 [file Table_1.DOCX]

**Table S1** League table for f1

| MD 95%CI | | | | | | |
| --- | --- | --- | --- | --- | --- | --- |
| 4_AP |  |  |  |  |  |  |
| -0.29 (-7.31, 6.7) | atomoxetine |  |  |  |  |  |
| -2.74 (-9.59, 4.09) | -2.45 (-5.03, 0.15) | L_Amphetamine |  |  |  |  |
| 2.9 (-123.08, 129.59) | 3.27 (-122.77, 129.84) | 5.72 (-120.3, 132.2) | Modafinil |  |  |  |
| 0.64 (-6.03, 7.34) | 0.93 (-1.18, 3.04) | 3.38 (1.9, 4.88)* | -2.3 (-128.86, 123.62) | Placebo |  |  |
| -1.12 (-8.18, 5.92) | -0.83 (-3.86, 2.23) | 1.62 (-1.02, 4.27) | -4.05 (-130.66, 121.92) | -1.76 (-3.95, 0.42) | Rivastigmine |  |
| -0.59 (-8.46, 7.29) | -0.3 (-4.93, 4.32) | 2.15 (-2.24, 6.51) | -3.53 (-130.13, 122.54) | -1.23 (-5.36, 2.87) | 0.53 (-4.13, 5.2) | simvastatin |

^* means p<0.05^
