# Supplementary material for: Pharmacological treatment options for cognitive dysfunction induced by multiple sclerosis: a network meta-analysis
Source: Front Neurol. 2025 Oct 7;16:1649429. doi: 10.3389/fneur.2025.1649429 (PMC12537379; doi:10.3389/fneur.2025.1649429)
Supplement: Supplementary file 9 [file Table_2.DOCX]

**Table S2** League table for f7

| MD 95%CI | | | | | | | | | | |
| --- | --- | --- | --- | --- | --- | --- | --- | --- | --- | --- |
| 4_AP |  |  |  |  |  |  |  |  |  |  |
| -3.53 (-15.04, 7.96) | Donepezil |  |  |  |  |  |  |  |  |  |
| 2.13 (-2.88, 7.1) | 5.67 (-4.89, 16.29) | Fampridine_SR |  |  |  |  |  |  |  |  |
| 1.72 (-3.1, 6.51) | 5.27 (-5.23, 15.78) | -0.41 (-1.84, 1.04) | Ginkgobiloba |  |  |  |  |  |  |  |
| 0.82 (-5.83, 7.46) | 4.36 (-7.08, 15.83) | -1.31 (-6.12, 3.51) | -0.9 (-5.54, 3.73) | L_Amphetamine |  |  |  |  |  |  |
| 4.42 (-3.54, 12.35) | 7.97 (-4.41, 20.24) | 2.28 (-4.22, 8.83) | 2.69 (-3.68, 9.08) | 3.59 (-4.26, 11.45) | Melatonin |  |  |  |  |  |
| -4.37 (-10.32, 1.55) | -0.83 (-11.91, 10.28) | -6.49 (-10.28, -2.72)* | -6.1 (-9.63, -2.57)* | -5.19 (-11, 0.59) | -8.78 (-16.09, -1.5)* | Memantine |  |  |  |  |
| -4.26 (-13.42, 4.92) | -0.72 (-13.79, 12.38) | -6.39 (-14.25, 1.53) | -5.97 (-13.76, 1.82) | -5.05 (-14.12, 3.95) | -8.66 (-18.75, 1.35) | 0.12 (-8.43, 8.68) | Methylphenidate |  |  |  |
| 1.63 (-3.19, 6.41) | 5.17 (-5.33, 15.68) | -0.5 (-1.9, 0.9) | -0.1 (-0.43, 0.24) | 0.8 (-3.81, 5.42) | -2.78 (-9.16, 3.57) | 6 (2.49, 9.53)* | 5.88 (-1.9, 13.66) | Placebo |  |  |
| -0.08 (-5.84, 5.61) | 3.46 (-7.48, 14.41) | -2.21 (-5.6, 1.21) | -1.81 (-4.94, 1.34) | -0.91 (-6.48, 4.67) | -4.5 (-11.59, 2.59) | 4.28 (-0.42, 9) | 4.17 (-4.26, 12.55) | -1.71 (-4.82, 1.41) | Rivastigmin |  |
| -2.26 (-8.66, 4.17) | 1.26 (-10.08, 12.66) | -4.39 (-8.87, 0.13) | -3.99 (-8.25, 0.3) | -3.08 (-9.34, 3.2) | -6.67 (-14.33, 1) | 2.11 (-3.42, 7.65) | 1.99 (-6.88, 10.86) | -3.89 (-8.15, 0.39) | -2.17 (-7.44, 3.1) | simvastatin |

^* means p<0.05^
