# Supplementary material for: Pharmacological treatment options for cognitive dysfunction induced by multiple sclerosis: a network meta-analysis
Source: Front Neurol. 2025 Oct 7;16:1649429. doi: 10.3389/fneur.2025.1649429 (PMC12537379; doi:10.3389/fneur.2025.1649429)
Supplement: Supplementary file 10 [file Table_3.DOCX]

**Table S3** League table for f8

| MD 95%CI | | | | | | | | | | |
| --- | --- | --- | --- | --- | --- | --- | --- | --- | --- | --- |
| 4_AP |  |  |  |  |  |  |  |  |  |  |
| -7.61 (-11.64, -3.59)^*^ | atomoxetine |  |  |  |  |  |  |  |  |  |
| 0.05 (-39.4, 39.55) | 7.68 (-31.75, 47.05) | CorSeNs |  |  |  |  |  |  |  |  |
| 0.18 (-4.77, 5.15) | 7.8 (3.26, 12.29)^*^ | 0.1 (-39.44, 39.63) | Donepezil |  |  |  |  |  |  |  |
| 1.9 (-5.26, 9.06) | 9.51 (2.67, 16.38)^*^ | 1.82 (-38.01, 41.8) | 1.73 (-5.74, 9.17) | Ginkgobiloba |  |  |  |  |  |  |
| -0.07 (-5.25, 5.1) | 7.54 (2.77, 12.31)^*^ | -0.14 (-39.62, 39.46) | -0.24 (-5.82, 5.31) | -1.97 (-9.56, 5.6) | L_Amphetamine |  |  |  |  |  |
| -1.05 (-12.07, 9.96) | 6.56 (-4.32, 17.36) | -1.13 (-41.86, 39.71) | -1.24 (-12.46, 10.02) | -2.95 (-15.29, 9.29) | -0.99 (-12.32, 10.29) | lutein |  |  |  |  |
| -1.48 (-6.03, 3.09) | 6.14 (2.06, 10.22) | -1.51 (-41.11, 38.02) | -1.65 (-6.65, 3.34) | -3.37 (-10.55, 3.81) | -1.4 (-6.61, 3.83) | -0.41 (-11.44, 10.66) | Memantine |  |  |  |
| -1.88 (-6.4, 2.65) | 5.73 (1.7, 9.79)^*^ | -1.97 (-41.42, 37.59) | -2.05 (-7.01, 2.92) | -3.79 (-10.98, 3.39) | -1.8 (-6.98, 3.38) | -0.82 (-11.82, 10.21) | -0.4 (-4.97, 4.17) | Modafinil |  |  |
| -1.08 (-4.28, 2.11) | 6.53 (4.07, 8.99)^*^ | -1.15 (-40.48, 38.24) | -1.26 (-5.06, 2.54) | -2.99 (-9.38, 3.41) | -1.01 (-5.1, 3.07) | -0.04 (-10.57, 10.53) | 0.39 (-2.86, 3.64) | 0.79 (-2.41, 3.99) | Placebo |  |
| 0.32 (-4.46, 5.13) | 7.93 (3.62, 12.26)^*^ | 0.28 (-39.26, 39.91) | 0.14 (-5.07, 5.37) | -1.58 (-8.88, 5.74) | 0.39 (-5.01, 5.8) | 1.38 (-9.73, 12.58) | 1.79 (-3.03, 6.61) | 2.2 (-2.6, 7) | 1.4 (-2.14, 4.97) | Rivastigmine |

^* means p<0.05^
