# Supplementary material for: Pharmacological treatment options for cognitive dysfunction induced by multiple sclerosis: a network meta-analysis
Source: Front Neurol. 2025 Oct 7;16:1649429. doi: 10.3389/fneur.2025.1649429 (PMC12537379; doi:10.3389/fneur.2025.1649429)
Supplement: Supplementary file 11 [file Table_4.DOCX]

**Table S4** League table for f13

| MD 95%CI | | | | | |
| --- | --- | --- | --- | --- | --- |
| atomoxetine |  |  |  |  |  |
| 2.83 (-2.07, 7.76) | CorSeNs |  |  |  |  |
| 4.99 (1.97, 8.02)^*^ | 2.16 (-1.77, 6.08) | Ginkgobiloba |  |  |  |
| 4.47 (0.89, 8.07)^*^ | 1.64 (-2.73, 5.98) | -0.52 (-2.52, 1.49) | L_Amphetamine |  |  |
| 4.27 (0.89, 7.66)^*^ | 1.44 (-2.75, 5.64) | -0.71 (-2.34, 0.89) | -0.2 (-2.7, 2.3) | Memantine |  |
| 4.97 (1.98, 7.98)^*^ | 2.14 (-1.76, 6.04) | -0.01 (-0.43, 0.4) | 0.5 (-1.47, 2.46) | 0.7 (-0.85, 2.26) | Placebo |

^* means p<0.05^
