# Supplementary material for: Pharmacological treatment options for cognitive dysfunction induced by multiple sclerosis: a network meta-analysis
Source: Front Neurol. 2025 Oct 7;16:1649429. doi: 10.3389/fneur.2025.1649429 (PMC12537379; doi:10.3389/fneur.2025.1649429)
Supplement: Supplementary file 12 [file Table_5.DOCX]

**Table S5** League table for f5 nausea

| OR 95%CI | | | | |
| --- | --- | --- | --- | --- |
| 4_AP |  |  |  |  |
| 20 (1.07, 890.36)^*^ | atomoxetine |  |  |  |
| 3.81 (0.54, 31.74) | 0.2 (0.01, 2.01) | Donepezil |  |  |
| 3.06 (0.5, 22.81) | 0.16 (0.01, 1.43) | 0.81 (0.41, 1.59) | Placebo |  |
| 1.94 (0.25, 17.5) | 0.1 (0, 1.11) | 0.51 (0.16, 1.58) | 0.63 (0.25, 1.55) | Rivastigmine |

^* means p<0.05^
