# Supplementary material for: Pharmacological treatment options for cognitive dysfunction induced by multiple sclerosis: a network meta-analysis
Source: Front Neurol. 2025 Oct 7;16:1649429. doi: 10.3389/fneur.2025.1649429 (PMC12537379; doi:10.3389/fneur.2025.1649429)
Supplement: Supplementary file 13 [file Table_6.DOCX]

**Table S6** League table for f5-insomnia

| OR 95%CI | | | | |
| --- | --- | --- | --- | --- |
| 4_AP |  |  |  |  |
| 1.25 (0.07, 22.29) | atomoxetine |  |  |  |
| 1.3 (0.09, 16.96) | 1.04 (0.09, 10.81) | Donepezil |  |  |
| 0 (0, 1.93) | 0 (0, 1.44) | 0 (0, 1.28) | L_Amphetamine |  |
| 0.39 (0.04, 2.95) | 0.32 (0.04, 1.72) | 0.3 (0.06, 1.15) | 17006.58 (0.27, 492340321237311) | Placebo |
|  |  |  |  |  |

^* means p<0.05^
