# Supplementary material for: Pharmacological treatment options for cognitive dysfunction induced by multiple sclerosis: a network meta-analysis
Source: Front Neurol. 2025 Oct 7;16:1649429. doi: 10.3389/fneur.2025.1649429 (PMC12537379; doi:10.3389/fneur.2025.1649429)
Supplement: Supplementary file 14 [file Table_7.DOCX]

**Table S7** League table for indigestion

| OR 95%CI | | |
| --- | --- | --- |
| Donepezil |  |  |
| 3.91 (1, 20.22) | Placebo |  |
| 0.38 (0.01, 5.19) | 0.1 (0, 0.73)^*^ | Rivastigmine |

^* means p<0.05^
